# Reconfigurable Light Field Generation With Hologram‐Enhanced Multicore Fibers

**DOI:** 10.1002/nap2.70332

**Published:** 2026-10-19

**Authors:** Mohammadhossein Khosravi, Leonidas Zbinden, Bennet Fischer, Adrian Lorenz, Jian Kim, Robert Leitel, Zhirong Tao, Carsten Rockstuhl, Thomas Sturges, Markus Nyman, Markus A. Schmidt

**Affiliations:** ^1^ Leibniz Institute of Photonic Technology Jena Germany; ^2^ Abbe Center of Photonics and Faculty of Physics Friedrich‐Schiller‐University Jena Jena Germany; ^3^ Fraunhofer‐Institute for Applied Optics and Precision Engineering IOF Jena Germany; ^4^ Institute of Theoretical Solid State Physics Karlsruhe Institute of Technology Karlsruhe Germany; ^5^ Institute of Nanotechnology Karlsruhe Institute of Technology Karlsruhe Germany; ^6^ Center for Integrated Quantum Science and Technology (IQST) Karlsruhe Institute of Technology Karlsruhe Germany; ^7^ Otto Schott Institute of Materials Research (OSIM) Friedrich Schiller University Jena Jena Germany

**Keywords:** 3D nanoprinting, metafiber, multicore fiber, phase‐only hologram, structured light

## Abstract

Tailoring light fields at remote or confined locations is a key requirement for many emerging photonic technologies. Here, a multicore fiber functionalized with a 3D‐printed hologram for the reconfigurable generation of structured light distributions is demonstrated. A computationally optimized 3D‐printed phase‐only hologram maps the fields emitted by the individual fiber cores onto prescribed output field distributions. By exciting different combinations of cores, these distributions can be combined into various reconfigurable light patterns. The concept is demonstrated by generating seven‐segment digits, which clearly illustrate the possibility of programmable pattern formation. The hologram is fabricated by high‐resolution 3D nanoprinting on a spacer integrated with the fiber facet, enabling beam expansion before wavefront shaping. Systematic optical characterization confirms reliable image formation and shows that the generated patterns are robust to variations in the relative phases between cores. The approach provides a compact and remotely addressable route to structured illumination, with potential applications in life sciences, telecommunications, and integrated photonics.

## Graphical abstract

A novel metafiber platform enables reconfigurable generation of structured light patterns directly at the end of a multicore optical fiber. A 3D‐printed hologram maps individual fiber cores onto predefined image elements, allowing programmable pattern synthesis through selective core excitation. The concept provides a compact route toward remotely addressable structured illumination for applications in life sciences, telecommunications, and integrated photonics.

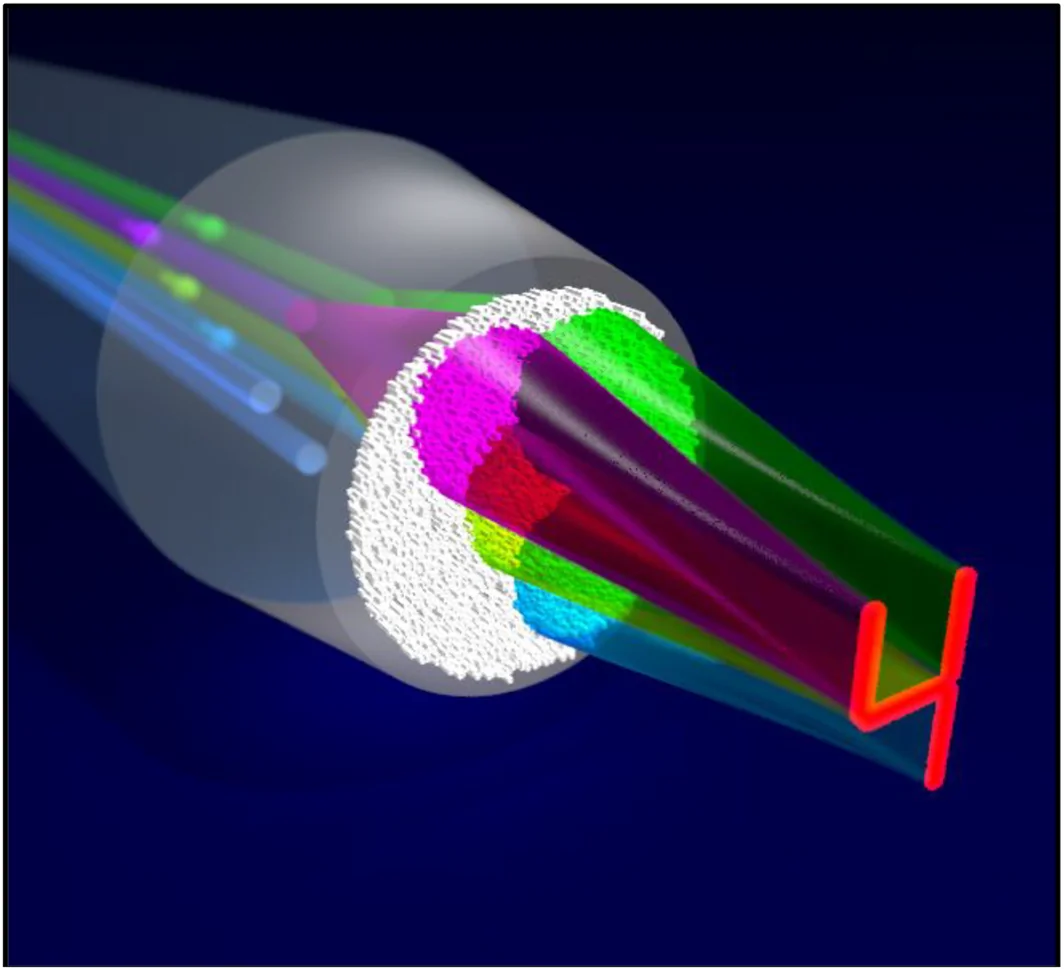

## 1 Introduction

The generation of complex light field distributions has emerged as a highly attractive research area from both fundamental and applied perspectives, with strong relevance to quantum technologies [1], life sciences [2], and advanced optical manipulation [3].

A particularly compelling approach to generating complex light distributions involves dielectric nanostructures [4], with a primary focus on metasurfaces and phase‐only holograms. Although metasurfaces, that is, arrays of subwavelength elements, engineered to tailor the phase and amplitude of reflected or transmitted light, enable multimodal beam shaping [5, 6], their fabrication complexity and limited diffraction efficiency pose significant challenges. In contrast, phase‐only holograms offer an efficient solution for phase and intensity modulation, featuring straightforward designs that enhance mechanical stability, reduce costs, and improve diffraction efficiency. These structures have been successfully implemented in applications such as optical tweezers [7], augmented reality [8], and metrology [9].

Most existing implementations use glass substrates in bulk optical setups, which limit their use in applications requiring remote and flexible light control. Here, optical fibers provide an excellent solution for efficient light transport to target locations. Having revolutionized fields, such as telecommunications [10], quantum technologies [11], and sensing [12], optical fibers represent a promising platform for remote light control when interfaced with nanostructures.

The integration of planar nanophotonic structures with optical fibers has given rise to the field of metafibers, in which functional nanostructures, such as metasurfaces or holograms, are directly fabricated on the end facet of an optical fiber to shape the optical field emitted by the fiber. A key driver of metafiber research has been the advent of 3D nanoprinting. Unlike conventional lithography, 3D nanoprinting is compatible with fiber geometries and allows independent control of the height of each structural element [13, 14]. Noteworthy advancements in metafiber research include optical tweezers [15], phase‐controlled multifocal arrays [16], metasurface‐based achromatic focusing [17], and complex beam shaping [18] as well as architectures designed to enhance fiber coupling [19, 20].

Although metafibers have greatly expanded the application domain of remote structured light generation, the static nature of the resulting distributions remains a limiting factor. Reconfigurable intensity distributions are required, for instance, in high‐resolution imaging [21], tissue scanning [22], or high‐capacity telecommunications [23], with recent metasurface approaches demonstrating advanced polarization and spatial‐channel multiplexing [24, 25]. This limitation underscores the need to expand the capabilities of the underlying fiber platforms.

Multicore fibers (MCFs) with single‐mode cores and suppressed intercore crosstalk have emerged as a key research topic in modern fiber optics [26], addressing challenges such as increased telecommunication data rates [27], power scaling of fiber lasers [28, 29], and advanced biomedical imaging [30]. Using MCFs in the context of metafibers offers a powerful platform for reconfigurable remote beam control. Initial demonstrations include tunable focal spot generation via on‐fiber holograms [31, 32], and angular demultiplexing using periodic nanostructures [33]. These results highlight the strong potential of multicore metafibers for remote and dynamically reconfigurable light field generation.

In this work, we present a multicore metafiber platform for reconfigurable generation of structured light distributions using a nanostructure‐functionalized MCF. The approach relies on a deterministic mapping between individual fiber cores and distinct image elements in the far‐field, realized through a computationally optimized phase‐only hologram fabricated by 3D nanoprinting on a spacer‐enhanced MCF (Figure 1). As a proof of principle, selective core excitation and phase control are used to synthesize reconfigurable seven‐segment digit patterns at a defined distance from the fiber end face. The ability to generate reconfigurable patterns provides a versatile platform for remotely addressable light‐shaping in confined and hard‐to‐access environments, with applications in life sciences, telecommunications, and integrated photonics.

**FIGURE 1:**
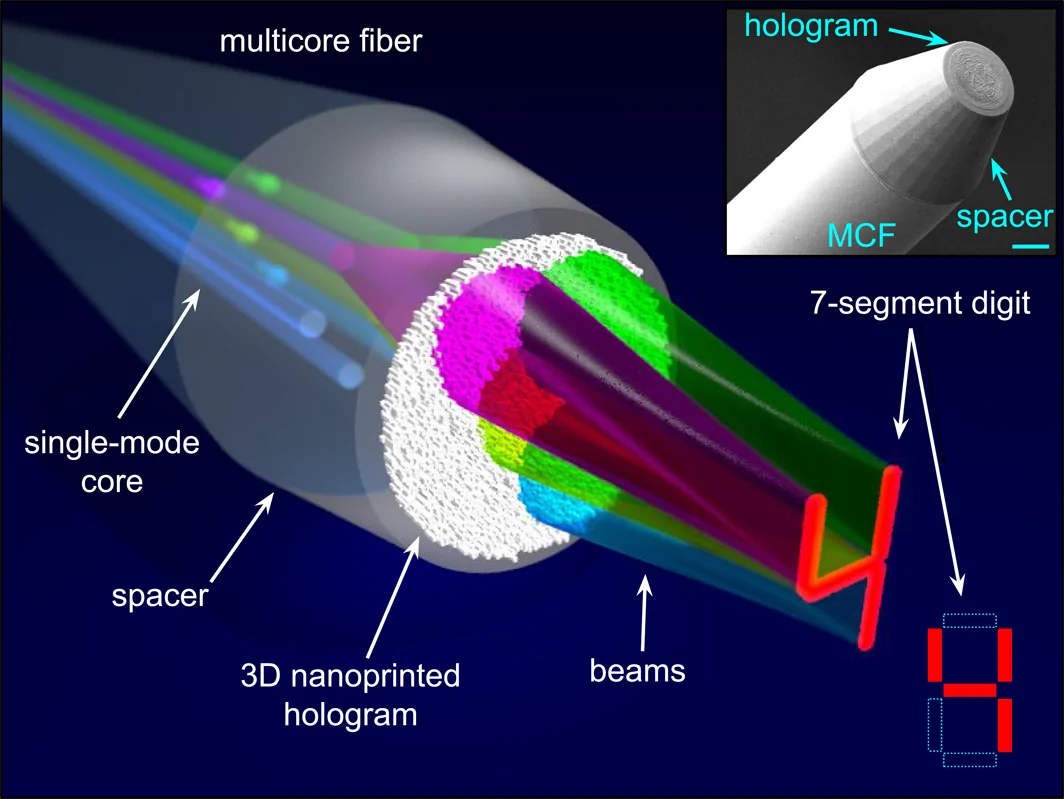
Artistic view of the concept of reconfigurable light‐field generation using a hologram‐enhanced multicore fiber, exemplified in this work by the generation of a seven‐segment digit pattern (light blue: MCF, semitransparent gray: spacer, white: 3D nanoprinted hologram, and colored regions: emitted beams). Top right inset: SEM image of the fabricated spacer‐enhanced MCF with the nanoprinted hologram on the end facet (scale bar: 50 μm). Bottom right inset: example of a generated digit (indicated by the red bars), with dashed lines indicating elements not used for the displayed number.

## 2 Concept

The objective of this work is to generate reconfigurable and structured light fields using a phase‐only hologram 3D‐printed directly onto the facet of a multicore fiber (Figure 2a). The central design principle is to establish an unambiguous correspondence between individual fiber cores and distinct far‐field image elements (Figure 2b). This deterministic core‐to‐image correspondence enables tailored intensity patterns by selectively exciting one or more cores, as demonstrated here by synthesizing digit‐like far‐field patterns analogous to a seven‐segment display.

**FIGURE 2:**
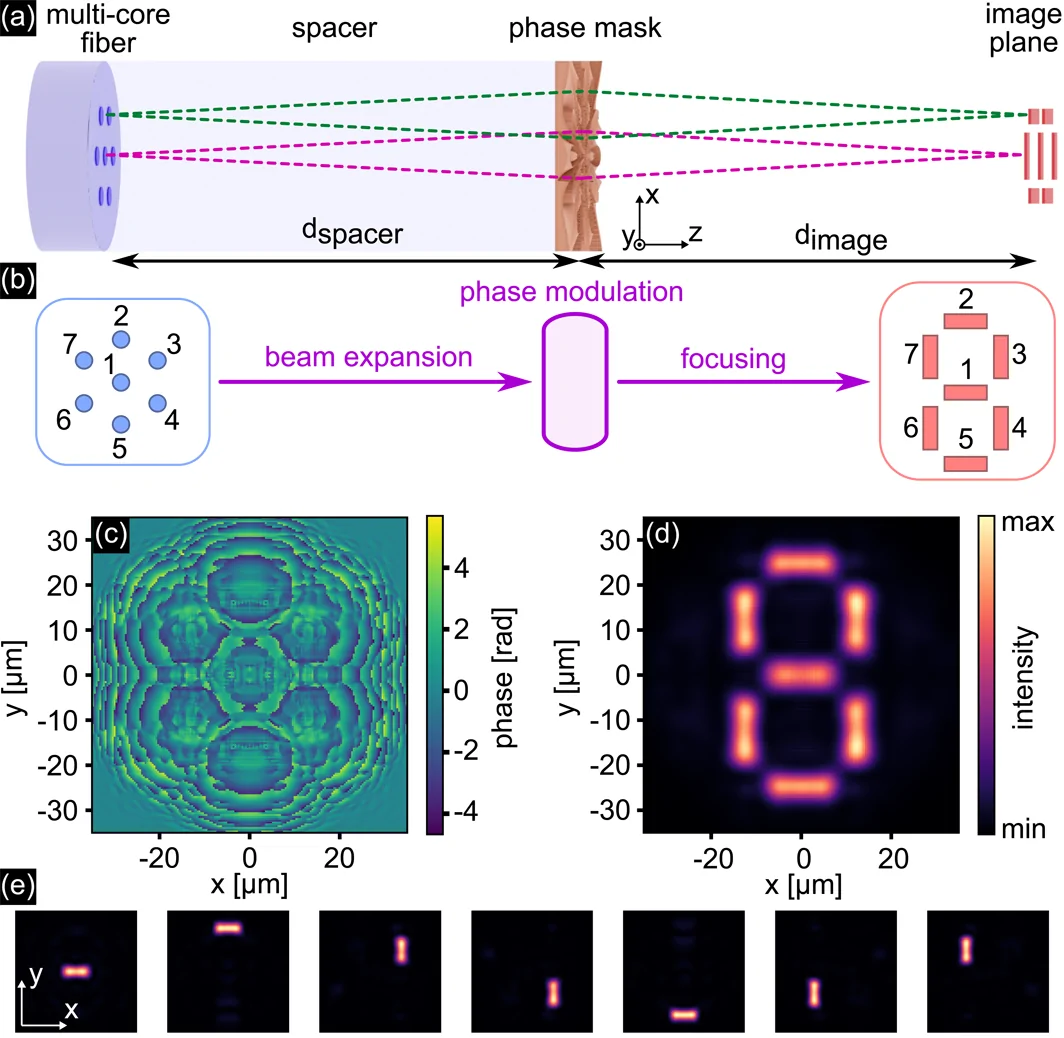
Concept and numerical demonstration of the multicore metafiber image‐generation principle. (a) Schematic of the system consisting of MCF, spacer (length: $d_{\mathrm{spacer}}$) phase mask, and the image plane (located at distance $d_{\mathrm{image}}$). Dashed lines illustrate the propagation of selected Gaussian beams emitted from individual fiber cores. (b) Mapping between the MCF geometry and the seven‐segment image layout. Individual cores (numbered 1–7, inset with a light blue frame) are deterministically assigned to specific image segments (inset with a light red frame). The light evolution involves beam expansion, phase modulation by the hologram and focusing to form the desired pattern. (c) Optimized phase profile of the optimized hologram. (d) Simulated intensity distribution at the image plane when all cores are excited in phase, synthesizing the digit “8”. (e) Simulated intensity distributions at the image plane when single cores are excited individually, demonstrating generation of spatially localized and independently addressable image elements.

We consider a single‐mode MCF comprising seven cores arranged in a hexagonal geometry (one central core surrounded by six satellite cores). The intercore distance is Λ = 14.6 μm, with each core having a numerical aperture NA = 0.08 at the operating wavelength $\lambda_{0}$ = 637 nm. This configuration ensures negligible intermodal coupling between cores [26] and allows the emitted field from each core to be well approximated by a Gaussian beam ($w_{0}$ = 2.8 μm). The spreading of these Gaussian beams is illustrated by the dashed lines; at the position of the hologram, adjacent beams overlap slightly. The hologram is designed to direct the light from a particular fiber core (inset with a light blue frame in Figure 2b) to a specific segment in the image (inset with a light red frame in Figure 2b). The image segments correspond to specific bars of a seven‐segment digit (width: 5 μm and length: 15 μm) as indicated by the numbers in Figure 2b.

The hologram design procedure relies on an inverse design technique based on automatic differentiation of a Fourier‐optics‐based simulation code [34]. The computational domain is discretized using a regular two‐dimensional grid, in which the transmission phase at each hologram grid point is treated as an independent optimization parameter, yielding a free‐form phase profile (see the Method section for details). An objective function is formulated and maximized using a local gradient‐descent‐based optimizer. This objective function comprises three contributions:

1. Maximization of the overlap between the individual fiber‐core intensity distributions at the image plane and their respective target patterns,
2. Maximization of the overlap with the intensity distribution corresponding to the digit 8 under simultaneous excitation of all cores, thereby accounting for interference effects, and
3. Minimization of intensity variations among the individual segments.

The full mathematical formulation of the objective function and the relevant computational parameters are provided in the Methods section.

The transmission phase profile of the designed hologram (Figure 2c) reflects the underlying hexagonal core arrangement, with the simulated intensity distribution at the image plane under simultaneous excitation of all cores (Figure 2d) showing the anticipated image. The target digit eight is clearly formed, with slightly blurred segment edges resulting from the finite numerical aperture of the system. Minor intensity inhomogeneities along the segments arise from partial overlap among the individual beams, which limits the ability to address the different holograms completely independently. The intensity distributions obtained under single‐core excitation (Figure 2e) are well confined and spatially separated, enabling the realization of all 10 digits and, more generally, any of the 128 possible segment combinations.

## 3 Implementation and Experiments

The MCF (outer diameter: 200 μm) was fabricated using the stack‐and‐draw method. It consists of 37 $GeO_{2}$‐doped silica cores with a diameter of 4.4 μm, arranged in a hexagonal silica lattice and operating in the single‐mode regime at $\lambda_{0}$ = 637 nm (Figure 3a). As reported in Ref. [26], the cores exhibit excellent uniformity with an identical numerical aperture of NA = 0.08. Note that in the present study, only the central core and the innermost satellite cores are addressed, resulting in an effective intercore pitch of 14.6 μm.

**FIGURE 3:**
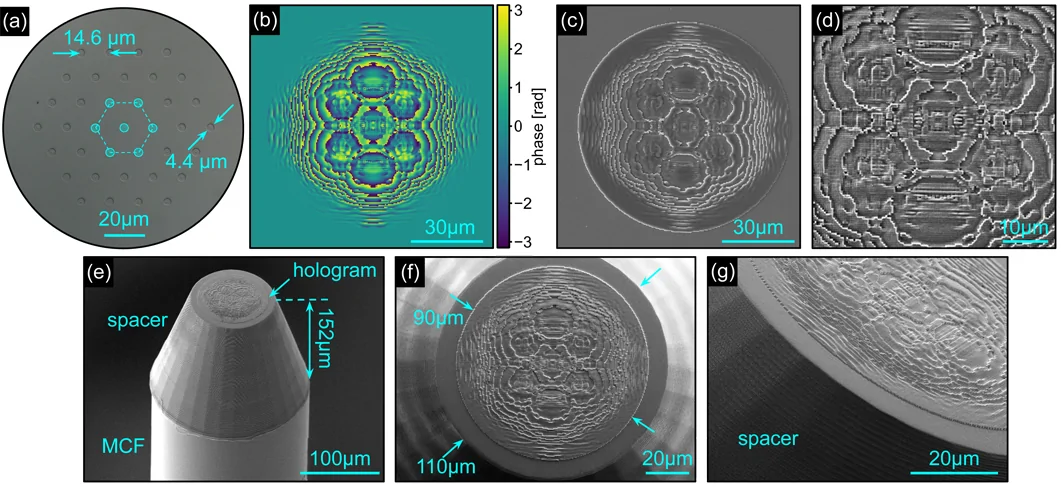
Structural characterization of the implemented multicore metafiber. (a) Microscopic image of the MCF end facet without nanostructure (intercore pitch: 14.6 μm and core diameter: 4.4 μm). The semitransparent cyan circles mark the cores used to generate the seven‐segment digit. (b) Designed phase pattern of the hologram. (c) and (d) Reference holograms printed on planar substrates. (e) Tilted SEM view of the complete device, showing the cone‐shaped polymer spacer printed on the MCF end facet with the hologram on top. (f) Top‐view SEM image of the on‐fiber hologram, illustrating precise alignment with the spacer center. (g) Magnified side view highlighting the fine structural features of the hologram and the spacer.

Implementation of the hologram, including the spacer, on the end face of the MCF, relied on 3D nanoprinting, carried out here by femtosecond direct laser writing using a commercial system (Photonic Professional GT2, Nanoscribe GmbH; see Methods for details). In this process, a tightly focused femtosecond laser beam induces two‐photon polymerization within a photosensitive resist, allowing polymerization to be spatially confined to the focal volume. By scanning the laser focus through the resist in three dimensions, the desired polymer structure is directly written onto the fiber end face. In contrast to conventional planar lithographic techniques, which are typically optimized for wafer‐based fabrication, this direct‐writing approach is highly compatible with the geometry of optical fiber end faces. Note that the hologram performance is strongly influenced by the voxel dimensions (transverse direction ≈ 300 nm and axial direction ≈ 700 nm), which determine the achievable spatial resolution. To enhance adhesion and prevent delamination of the hologram, the MCF facet is plasma‐treated and silanized before printing. Before fabricating the holographic structures, a cone‐like polymer spacer (length, $L_{s}$ = 150 μm) was printed onto the MCF facet to enable beam expansion.

Mode excitation of multiple cores simultaneously was achieved using a custom‐built microscopic setup using a HeNe laser operating at 632.8 nm that generates a desired number of focused spots in the same plane. Note that the hologram was optimized for $\lambda_{0}$ = 637 nm; however, the resulting wavelength difference of less than 1% leads to only negligible changes in the polymer refractive index and phase modulation and therefore does not affect the generated patterns. Via imaging the MCF input facet, light was coupled into specific cores by using a spatial light modulator (SLM) in combination with an objective lens (details can be found in the Method section and in Supporting Information S1: Section S1). This setup enables independent control of the relative amplitudes and phases of the emitted light spots, with a coupling efficiency of ≈ 40% (see definition in Supporting Information S1: Section S2), as confirmed by multiple consistency checks performed prior to the experiments. The achieved coupling efficiency of ≈ 40% represents a comparatively high value considering the complexity of the optical setup required for selective and reconfigurable multicore excitation.

The intensity characterization setup included both illumination and imaging paths. An LED illuminated the 3D‐printed fiber endface through relay optics and an objective lens, enabling identification and focusing on the fiber surface. The reflected light was collected by the same objective lens and imaged onto a CMOS camera via a tube lens. In addition, the same optical path was used to image the intensity distributions generated in the image plane onto the CMOS camera. Auxiliary illumination and alignment paths were integrated to ensure reproducible excitation and detection conditions (Supporting Information S1: Section S1).

## 4 Results

Representative SEM images of the fabricated device, including the hologram on the MCF end facet, the corresponding design, and reference holograms printed on planar substrates, are shown in Figure 3. The high structural fidelity of the on‐fiber hologram is evident (Figure 3e–g). It accurately reproduces both the coarse and fine features of the designed phase pattern (Figure 3b) and closely matches the trial structures fabricated on planar substrates (Figure 3c,d). The hologram is precisely aligned with the center of the spacer (Figure 3f). The spacer exhibits a characteristic cone‐like geometry (Figure 3e), which ensures mechanical stability while providing sufficient beam expansion.

Topographic characterization of reference holograms fabricated on planar substrates further confirms the high fidelity of the fabrication process (see Supporting Information S1: Section S3). Laser‐scanning microscopy measurements show good agreement between the fabricated surface relief and the design, reproducing both the overall geometry and the fine structural features relevant to beam shaping. Slight deviations in absolute feature heights and slight smoothing of sharp transitions are attributed to fabrication‐related factors, including material shrinkage, exposure‐dose variations, finite voxel dimensions, and the intrinsic low‐pass filtering inherent in two‐photon polymerization. Importantly, the relative arrangement of the surface features remains well preserved, indicating that the phase relationships governing beam shaping are largely maintained.

To verify the operating principle for generating bar‐like light distributions required for a seven‐segment display, light was sequentially coupled into individual cores, and the resulting intensity distributions in the image plane were recorded with a camera positioned at the target distance of 150 μm behind the hologram (Figure 4). The measurements confirm that each selectively excited core produces a distinct, spatially separated bar‐like pattern at the target plane. The overall shape and position of the generated segments agree well with the design concept. Slight deviations from the ideal bar geometry appear in the form of minor asymmetries, intensity inhomogeneities, and weak background modulations. Such deviations can be attributed to fabrication tolerances in the 3D nanoprinted hologram, finite voxel resolution, and small alignment offsets between the cores and the hologram. The experimentally determined diffraction efficiency of a single generated bar reaches 18.4%, representing a solid value for a free‐form, on‐fiber phase hologram and confirming efficient light utilization of the design (see Supporting Information S1: Section S3 for details of the evaluation procedure). Applying the same evaluation procedure to the simulated intensity distribution yields a diffraction efficiency of ≈ 38%. Therefore, the experimentally achieved value corresponds to ≈ 50% of the simulated efficiency, with the difference attributed primarily to fabrication‐related deviations from the ideal hologram and experimental imperfections. Note that these imperfections do not compromise the functionality of the concept as the generated patterns remain clearly distinguishable and sufficiently well‐defined for reliable segment addressing.

**FIGURE 4:**
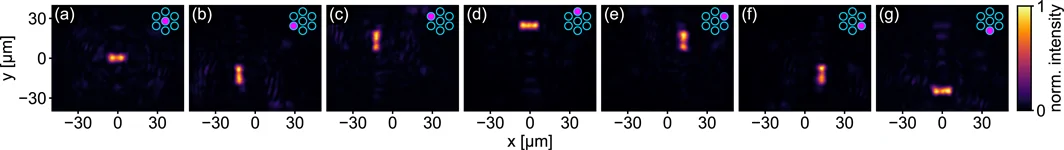
Single‐element light field generation via single‐core excitation. Spatial intensity distributions in the image plane, measured at a distance of 150 μm from the hologram in case of selective excitation of individual cores. Each panel corresponds to coupling into a different core, indicated by the magenta‐highlighted core in the schematic shown in the upper right corner of each image. The intensity in each panel is normalized to its respective maximum value, and the color scale is linear.

To further explore the multicore metafiber concept for digit generation, light was simultaneously coupled into selected combinations of cores to synthesize the digits 0–9 using the same experimental setup (Figure 5). The amplitude of each element was independently adjusted via amplitude modulation on the SLM to ensure approximately uniform intensity distribution across each formed digit as verified by monitoring the intensity profile on the camera. During this amplitude‐balancing procedure, no phase correction or phase modulation was applied to the excitation beams. All 10 digits are clearly recognizable, demonstrating that reconfigurable structured light patterns can be generated through appropriate coupling. As in the single‐core experiments (Figure 4), minor deviations from the ideal segment geometry are observed, including moderate intensity variations between segments, slight edge distortions, and weak background artifacts arising from similar mechanisms. These deviations do not compromise the readability or functionality of the generated digits as the numerical patterns remain distinguishable. In addition, z‐scan measurements around the design image plane confirm a well‐defined focus region with finite depth of field for all segments and robust digit visibility against moderate longitudinal displacement (Supporting Information S1: Figure S5).

**FIGURE 5:**
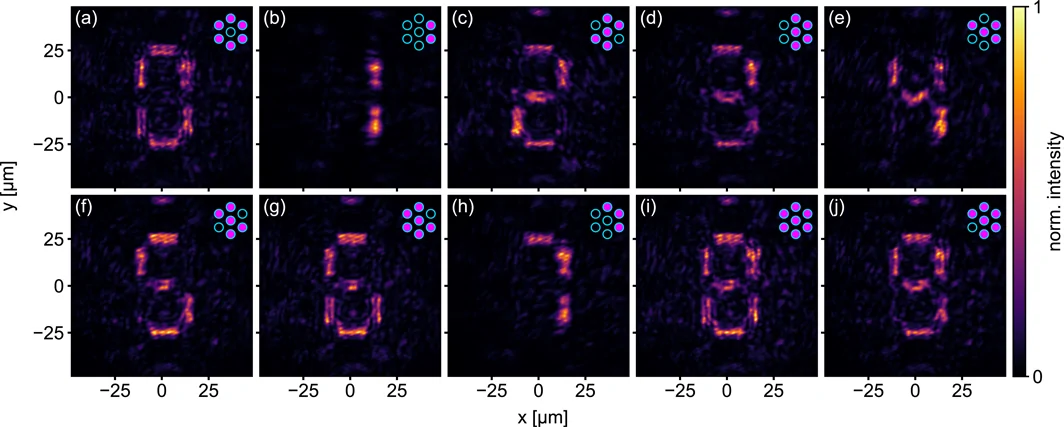
Reconfigurable digit generation via selective core excitation. Spatial intensity distributions in the image plane recorded at a distance of 150 μm from the hologram. (a–j) Each panel corresponds to a specific core excitation configuration that generates a different digit as indicated by the magenta‐highlighted cores in the schematic in the upper‐right corner of each image. The intensity in each panel is normalized to its respective maximum, and the color scale is displayed on a linear scale.

Due to the flexibility of SLM‐based light coupling, the relative phase of the light injected into the individual cores can be precisely controlled. This capability was used to enhance the readability of the synthesized digits by manually adjusting the intercore phases to optimize the appearance of the bar‐like intensity distributions (Figure 6). The resulting patterns exhibit higher segment contrast, improved brightness uniformity across the bars, and reduced background artifacts, yielding sharper, more clearly defined digits. As in the previous case, uniform intensity across each digit was ensured by monitoring the intensity distribution on the camera and independently adjusting the amplitude of each coupling beam generated by the SLM.

**FIGURE 6:**
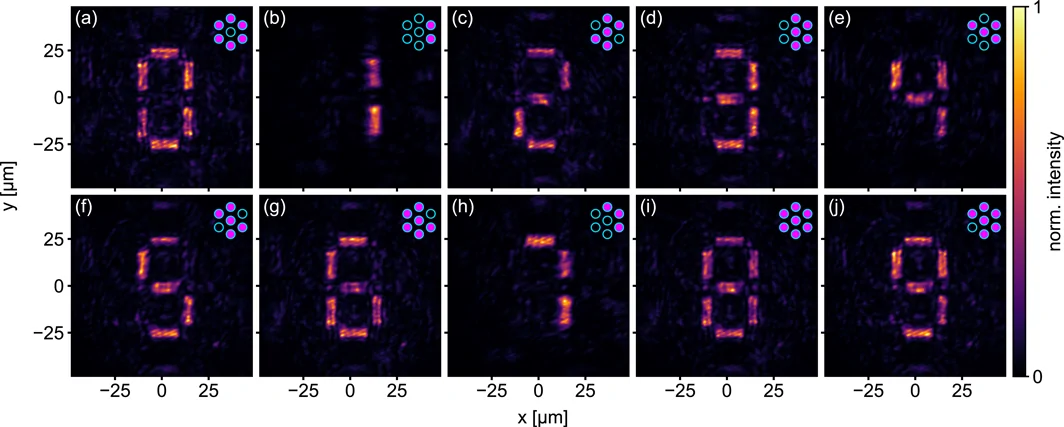
Digit synthesis via selective multicore excitation with intercore phase adjustment. Measured spatial intensity distributions in the image plane (normalized to the respective maximum value, linear scale) for different excitation configurations, including manual phase control between the excited cores. (a–j) Each panel corresponds to a specific combination of addressed cores generating a distinct digit, analogous to the configurations shown in Figure 5, with the excited cores indicated by magenta highlights in the schematic at the upper right of each image.

In the context of MCFs, one important aspect is their sensitivity to fiber bending. Bending generally introduces different optical path‐lengths for the various cores and thus modifies their relative phases, which can affect interference between the corresponding fields in the hologram plane. As described by McBride et al. [35], the bending‐induced phase difference between two cores is expected to increase approximately linearly with the bending angle. This behavior was experimentally quantified for the MCF used here, revealing a pronounced and approximately linear phase variation even for relatively small bending angles. Details of the bending experiment and phase‐retrieval procedure are provided in the Supporting Information S1: Section 6.

To emulate fiber bending, the phase of the beams exciting the cores in the MCF was randomly varied (0 < ΔΦ < 2π), and the resulting diffraction pattern in the image plane was recorded. The results, exemplified by the digit two in Figure 7, clearly demonstrate that the target digit remains recognizable across all applied random phase distributions, thereby showing distinct phase robustness.

**FIGURE 7:**
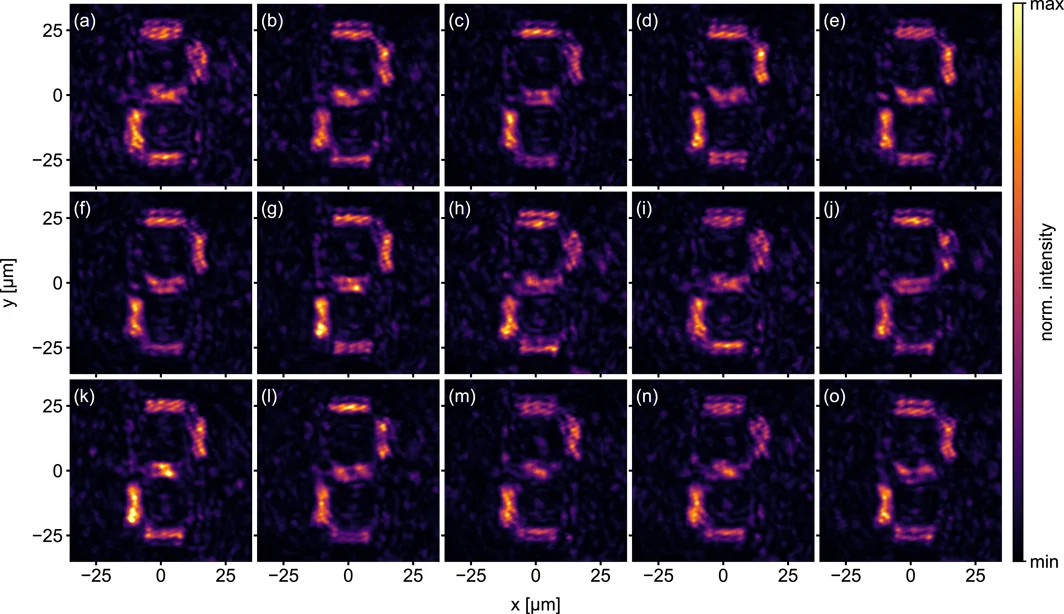
Synthesis of digit 2 via selective multicore excitation under random intercore phase distributions to emulate fiber bending. (a–o) Each panel shows the measured intensity distribution in the image plane for a distinct random phase configuration between the excited cores.

To quantitatively analyze the impact of phase variations on the generated light‐field patterns, a cross‐correlation analysis of the holographically reconstructed intensity distributions was performed. The purpose of this analysis is to quantify the robustness of the holographic reconstruction against random relative phase variations between the individual cores of the MCF. We specifically employ pairwise Pearson correlation analysis, whichprovides a quantitative measure of the similarity between the spatial intensity distributions obtained for different random phase realizations (see Supporting Information S1: Section S6).

Figure 8a shows the pairwise Pearson correlation matrix for seven representative experimental realizations selected from those shown in Figure 7. The relatively high off‐diagonal correlation coefficients demonstrate that the characteristic spatial intensity distribution is largely preserved for different random relative intercore phase configurations.

**FIGURE 8:**
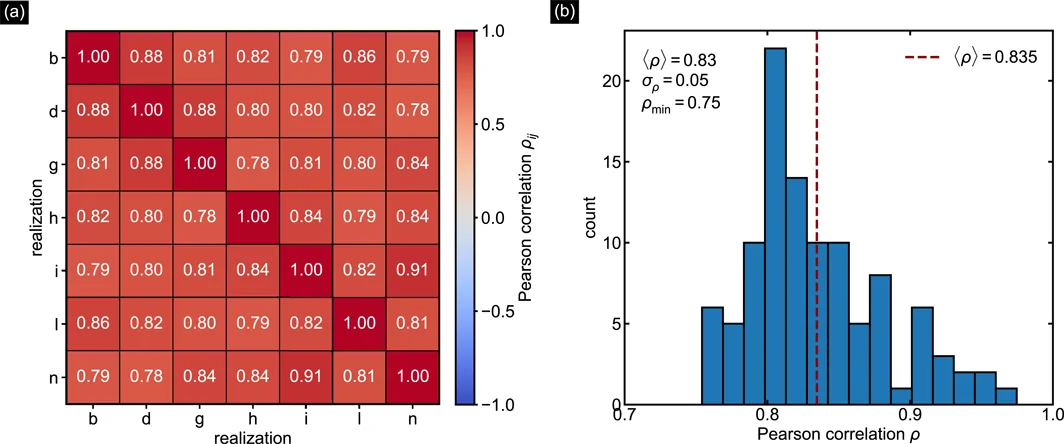
Quantitative analysis of the robustness of the experimentally generated intensity patterns against random relative inter‐core phase variations. (a) Pairwise Pearson correlation matrix for seven representative experimental realizations shown in Figure 7. (b) Histogram of the Pearson correlation coefficients obtained from all 105 pairwise comparisons between the 15 experimental realizations (Figure 7). The dashed line indicates the mean Pearson correlation coefficient of 〈ρ〉≈0.835. The distribution has a standard deviation of $\sigma_{\rho }\;\approx \;0.05$ and a minimum correlation coefficient of $\rho_{\min}\;\approx \;0.75$.

Furthermore, to obtain a more comprehensive statistical assessment, all 15 experimentally recorded realizations shown in Figure 7 were considered, corresponding to 105 independent pairwise comparisons. The resulting distribution of Pearson correlation coefficients is shown in Figure 8b. The coefficients exhibit a mean value of 〈ρ〉≈0.835, with a standard deviation of $\sigma_{\rho }\;\approx \;0.05$ and a minimum value of $\rho_{\min}\;\approx \;0.75$. Thus, even the least‐correlated pair of experimental realizations retains substantial similarity in its spatial intensity distribution, while most of the correlation coefficients remain within a relatively narrow range around the mean value. Overall, this quantitative analysis confirms the qualitative observation from Figure 7 that random relative phase variations between the individual MCF cores do not substantially alter the characteristic spatial distribution of the generated light field.

To further validate the applied phase control, supplementary measurements confirmed reliable phase transfer through the multicore fiber using two‐core interference experiments and demonstrated that the applied phase modulation does not affect the output intensity distribution, thereby confirming independent control of phase and amplitude (see Supporting Information S1: Sections S7 and S8).

## 5 Discussion

This work demonstrates the generation of multiple digits in the image plane through selective excitation of specific cores. Although digit formation remains visible for arbitrary intercore phase distributions, further improvements in phase robustness against fiber bending represent an important direction for future research. Advanced optimization strategies could enable hologram designs that are intrinsically phase‐insensitive, making the system largely bend‐insensitive. It should be emphasized that the experimentally determined optimized phase distribution (Figure 5) depends on the specific setup conditions. Minor variations, such as mechanical displacement of the fiber, objective lens, or spatial light modulator, require recalibration and renewed phase adjustment, clearly showing the need for a phase‐insensitive approach.

The present study focuses on spatially separated image elements in the target plane, which inherently excludes interference effects. A natural next step is the investigation of spatially overlapping intensity patterns, where interference becomes relevant and phase variations may play a critical role. This aspect is particularly important for generating continuous or fully resolved images beyond the bar‐like segment structures addressed in this work.

The multicore metafiber concept offers multiple directions for future research. These include generating more complex images with tailored phase control to excite resonant optical states such as fiber modes or nanophotonic resonances. Employing high‐core‐count imaging fibers, for example, Fujikura Imaging Fiber [36], would further increase the complexity of the patterns generated. Improved optimization algorithms and machine learning can further enhance hologram design [37], whereas advances in nanoprinting technologies, including STED‐based nanoprinting [38] and grayscale lithography [39], promise higher resolution and higher efficiency. Moreover, integrating polarization‐engineering metasurfaces provides additional degrees of freedom [18], enabling advanced on‐fiber beam manipulation.

Although digit generation serves as a proof‐of‐concept for the capabilities of the approach, the present work specifically demonstrates reconfigurable pattern generation through combinations of predefined image elements. Building on these demonstrated capabilities, several future application scenarios can be envisioned, particularly where reconfigurable light fields must be delivered to remote or confined locations. In life sciences, the configuration demonstrated in this work could be adapted to provide structured illumination patterns, such as stripes, checkerboards, or ring geometries, for contrast enhancement and computational microscopy through a single fiber. By extending the generated patterns beyond the bar‐like elements demonstrated here, future implementations could enable programmable optical‐trapping landscapes at the distal fiber tip and reconfigurable illumination for photoactivation and phototherapy, thereby allowing spatially controlled dose delivery in optogenetics, photodynamic therapy, or localized photochemistry. Through the use of specifically optimized holograms, controlled and homogeneous illumination profiles could further be generated for nanoparticle tracking and manipulation in microfluidic or biological environments.

In integrated photonics, holograms incorporating focusing and phase‐optimized functionalities could enable adaptive coupling into on‐chip devices by synthesizing mode‐matched input fields for efficient excitation of selected waveguide modes. Tailored phase and amplitude distributions could further be explored to excite resonant nanophotonic states, including nanoscale resonances or plasmonic antennas. In telecommunications, the metafiber concept could potentially be extended toward spatial division multiplexing by acting as a reconfigurable spatial mode converter or routing element. Beyond these areas, patterned light delivery at the fiber tip could potentially be used for localized photochemistry, polymerization, or microfabrication inside confined geometries.

## 6 Summary and Outlook

The reconfigurable generation of complex light‐field distributions at specified remote locations is crucial for numerous advanced applications. In this work, we have demonstrated reconfigurable synthesis of structured light patterns using a nanostructure‐augmented multicore fiber, enabled by a deterministic mapping between individual cores and distinct far‐field image elements. As a proof of concept, seven‐segment digits were generated via selective core excitation and phase control, highlighting the scalability and flexibility of the concept.

The study combines a novel hologram design strategy using advanced computational optimization with high‐fidelity 3D nanoprinting on a spacer‐enhanced fiber facet and comprehensive optical characterization. The fabricated structures exhibit robust image formation, including resilience to intercore phase variations. Overall, this approach establishes a versatile platform for reconfigurable, remotely addressable light shaping with strong potential for structured illumination in confined or hard‐to‐access environments, such as life sciences, telecommunications, and integrated photonics.

## 7 Materials and Methods

### 7.1 Computational Methods

The thin‐element approximation is employed to describe light transmission through the hologram, whereas the propagation of the optical field through the spacer and the free‐space region between the hologram and the image plane is modeled using the angular spectrum method. The implementation is based on the JAX Python package [40], which enables automatic differentiation and thus direct calculation of the gradient of the objective function regarding the design variables. In this case, the design variables correspond to the transmission phase values of the hologram at each point on its surface.

*Objective function*: The objective function is based on the normalized cross‐correlation (NCC) between the calculated intensity distributions I and the target intensity distributions T. The NCC between two discretized intensity distributions is defined here as the scalar operator

(1) $$I⋆T=\frac{{\text{Cov}}_{x,y}\left(I,T\right)}{\sqrt{{\text{Var}}_{x,y}\left(I\right){\text{Var}}_{x,y}\left(T\right)}+\epsilon }$$

where ϵ>0 ensures numerical stability. The covariance is given by

(2) $${\text{Cov}}_{x,y}\left(I,T\right)=\frac{1}{N^{2}}\sum_{x,y}\left[I\left(x,y\right)-\bar{I}\right]\left[T\left(x,y\right)-\bar{T}\right].$$

Here x and y are the discretized spatial coordinates, sampled at N points along each direction, and the overbar denotes the corresponding spatial average. As usual, Var(X)=Cov(X,X). The objective function is composed of an unweighted sum of four components,

(3) $$F=F_{\text{ncc}}+F_{\text{std}}+F_{\text{int}}+F_{\text{uni}}$$

given as

(4) $$F_{\text{ncc}}=\frac{1}{7}\sum_{a=1}^{7}I_{a}⋆T_{a}\;F_{\text{std}}=-\sqrt{{\text{Var}}_{a}\left(I_{a}⋆T_{a}\right)}$$

(5) $$F_{\text{int}}=I_{\text{int}}⋆T_{\text{int}}\;F_{\text{uni}}=-{\text{Var}}_{a}\left(P_{a}\right).$$

The component $F_{\text{ncc}}$ is the average NCC between the intensity distributions $I_{a}\left(x,y\right)={\left|E_{a}\left(x,y\right)\right|}^{2}$ and their respective targets $T_{a}\left(x,y\right)$, where $E_{a}$ is the electric field when only the a‐th fiber core is illuminated. The penalty term $F_{\text{std}}$ promotes comparable performance across all cores. Because these single‐core objectives do not account for interference during simultaneous excitation, $F_{\text{int}}$ regularizes against poor multicore performance and is evaluated for in‐phase excitation of all cores, with $I_{\text{int}}\left(x,y\right)={\left|\sum_{a=1}^{7}E_{a}\left(x,y\right)\right|}^{2}$. Finally, $F_{\text{uni}}$ penalizes variations in power among the seven core contributions, where $P_{a}=\sum_{x,y}I_{a}\left(x,y\right)$. All contributions to the objective function were applied with unit coefficients, and no separate optimization of the weights was performed.

*Implementation*: The angular spectrum method was implemented using discrete Fourier transforms on the computational grid, enabling the use of fast Fourier transform algorithms. To suppress artificial wrap‐around effects arising from the inherent periodicity of the discrete Fourier transform, propagation from the phase mask to the image plane was divided into five equal steps, with an artificial iris applied after each of the first four steps. The computational domain spanned 100μm in both transverse directions and was discretized using 251×251 grid points. The hologram phase was initialized uniformly as zero, corresponding to a flat phase. The objective function was maximized using the Adam algorithm implemented in the Optax library [41]. A learning rate of $5\times 10^{-3}$ and a maximum of 4000 iterations were used, with the remaining Adam parameters set to their default values. The optimization was terminated early when the absolute change in the objective function between successive iterations fell below $10^{-7}$. No explicit constraints were imposed on the phase variables.

### 7.2 Implementation of the Hologram on Single‐Mode Multicore Fiber

*Properties of the nanoprinting process*: The 3D nanoprinting of the polymer‐based hologram and spacer is performed using two‐photon lithography via femtosecond direct laser writing with a commercial system (Photonic Professional GT, Nanoscribe GmbH). This mask‐less fabrication technique is based on two‐photon absorption‐induced polymerization, forming subwavelength ellipsoidal building blocks, referred to as voxels, with typical dimensions of 300 nm × 700 nm. The method enables rapid, reliable, and cost‐efficient fabrication and allows direct integration of nanostructures onto optical fiber end facets without the need for additional postprocessing or cleanroom infrastructure.

*Fiber surface activation*: Before nanoprinting, the fiber facet was chemically activated in a two‐step process involving (i) oxygen plasma activation applied for 1 min (150 W), (ii) followed by silanization based on overnight immersion in 1% 3‐(trimethoxysilyl) propyl methacrylate (Sigma‐Aldrich 440,159) dissolved in ethanol and nitrogen drying. Before the first step, the fiber tip was cleaned by successive sonication in acetone, isopropyl alcohol, and distilled water (15 min each), followed by nitrogen drying.

*Nanoprinting*: To align the MCF within the nanoprinter, light was coupled into the cores from the distal side, enabling precise identification of the fiber position using a low‐magnification objective (Plan‐Apochromat 20*x*/0.85, Zeiss). Subsequently, a drop of the negative‐tone photoresist IP‐Dip (Nanoscribe GmbH) was applied to the fiber facet, and the translation stage was moved to predefined 3D coordinates under a high‐numerical‐aperture oil immersion objective (Plan‐Apochromat 63*x*/1.40 Oil DIC, Zeiss). After locating the fiber end face under high magnification, 3D nanoprinting was initiated by focusing the femtosecond laser beam into the photoresist, where two‐photon absorption locally induces polymerization within the focal volume. The desired three‐dimensional geometry was then generated by scanning this focal volume through the resist according to the predefined structural design. First, a spacer with a length of 150 μm was fabricated using an increased exposure dose to ensure mechanical stability. The hologram was then printed on top of the spacer (hatching distance: 50 nm, slicing distance: 100 nm), resulting in a total fabrication time of approximately 30 min. Optimal printing conditions were achieved with a laser power of 11.5 mW and a scan speed of 8000 μm/s. After exposure, the sample was developed in propylene glycol monomethyl ether acetate (PGMEA, Sigma‐Aldrich 484,431) for 25 min, followed by a 3 min rinse in methoxy‐nonafluorobutane (Novec 7100, 3M). Finally, the structure was rinsed with isopropyl alcohol prior to optical characterization.

### 7.3 Optical Characterization: Mode Excitation Setup

Excitation of modes in selected cores was achieved by directing light from a continuous‐wave He–Ne laser (Thorlabs HNL100LB, $\lambda_{0}$ = 632.8 nm) into a chosen core of the hologram‐enhanced MCF using a spatial light modulator based setup (details can be found in Supporting Information S1: Section S1). Excitation of a specific core was enabled by a custom microscope arrangement comprising an objective (Olympus Plan N 10*x*, NA = 0.25) and a relay lens (Thorlabs AC254‐300‐A‐ML). This configuration projects an image of the MCF input facet together with the incident laser spot onto a CMOS camera (Thorlabs DCC1545 M), allowing precise selection of the desired core by transverse displacement of the laser spot across the fiber surface.

### 7.4 Optical Characterization: Characterization of Intensity Distribution in the Image Plane

The intensity distribution in the focal plane was recorded using a custom‐built microscope consisting of an objective (Olympus UPlanFL N 20*x*, NA = 0.5) and a relay lens (Thorlabs AC254‐300‐A‐ML), coupled to a CMOS camera (Thorlabs DCC1545 M) (more details can be found in Supporting Information S1: Section S1).

## Author Contributions

**Mohammadhossein Khosravi:** conceptualization, methodology, data curation, investigation, validation, formal analysis, visualization, writing – original draft, writing – review and editing, resources. **Leonidas Zbinden:** methodology, investigation, resources, writing – review and editing. **Bennet Fischer:** writing – review and editing, formal analysis, methodology, supervision. **Adrian Lorenz:** methodology, investigation, writing – review and editing. **Jian Kim:** methodology, writing – review and editing. **Robert Leitel:** methodology, writing – review and editing. **Zhirong Tao:** methodology, software, formal analysis, writing – review and editing, visualization, investigation, validation, data curation. **Carsten Rockstuhl:** supervision, writing – review and editing, funding acquisition. **Thomas Sturges:** methodology, software, supervision, formal analysis, validation. **Markus Nyman:** software, methodology, formal analysis, supervision, writing – review and editing, validation. **Markus A. Schmidt:** conceptualization, methodology, supervision, funding acquisition, project administration, Resources, writing – original draft, writing – review and editing, investigation.

## Conflicts of Interest

The authors declare no conflicts of interest.

## Supporting information


Supporting Information S1

## Data Availability

The data supporting the results of this study are available from the corresponding authors upon reasonable request.
