# Supplementary material for: Reconfigurable Light Field Generation With Hologram‐Enhanced Multicore Fibers
Source: Nanophotonics. 2026 Oct 19;15(20):e70332. doi: 10.1002/nap2.70332 (PMC13678425; doi:10.1002/nap2.70332)
Supplement: Supplementary file 1 [file NAP2-15-e70332-s001.pdf]

Supplementary Information

Reconfigurable Light Field Generation with Hologram-Enhanced Multicore Fibers

Mohammadhossein Khosravi[1,2], Leonidas Zbinden[1,2], Bennet Fischer[1], Adrian Lorenz[1], Jian Kim[1,2], Robert Leitel[3], Zhirong Tao[4], Carsten Rockstuhl[4,5,6], Thomas Sturges[5], Markus Nyman[5] and Markus A. Schmidt[1,2,7,*]

[1]Leibniz Institute of Photonic Technology (Leibniz IPHT), Jena, Germany;
[2]Abbe Center of Photonics and Faculty of Physics, Friedrich Schiller University Jena, Jena, Germany;
[3]Fraunhofer Institute for Applied Optics and Precision Engineering IOF, Jena, Germany;
[4]Institute of Theoretical Solid State Physics, Karlsruhe Institute of Technology, Karlsruhe, Germany;
[5]Institute of Nanotechnology, Karlsruhe Institute of Technology, Karlsruhe, Germany;
[6]Center for Integrated Quantum Science and Technology (IQST), Karlsruhe Institute of Technology, Karlsruhe, Germany;
[7]Otto Schott Institute of Materials Research (OSIM), Friedrich Schiller University Jena, Jena, Germany
*markus-alexander.schmidt@uni-jena.de

Sec. S 1 Experimental setup

Supplementary Fig. S1 shows the complete experimental setup used for selective core excitation and image-plane characterization of the hologram-enhanced multicore fiber. It consists of a selective core excitation section (light yellow background), the multicore fiber (light green background), and the intensity characterization section (light purple background). The operation of the selected core excitation part is as follows: A continuous-wave He–Ne laser (Thorlabs HNL100LB, $\lambda_0 = 632.8$ nm) is first attenuated by a neutral-density (ND) filter and directed to the SLM via mirrors M1 and M2. The laser beam is expanded and relayed onto the SLM by lenses L1 and L2, ensuring efficient illumination of the active modulation area.

The phase-modulated beam reflected from the SLM is relayed through lens L3 and directed toward the coupling path via mirrors M3 and M4. An iris in the Fourier plane serves as a spatial filter, suppressing unwanted diffraction orders and selecting the desired modulation order produced by the SLM. The filtered beam is then focused onto the fiber facet through lens L4, beam splitters BS1 and BS2, and an objective lens (Olympus Plan N 10x, NA = 0.25), enabling efficient, controlled coupling into selected cores of the MCF. The same objective serves for both monitoring and alignment. Auxiliary illumination from LED1 is coupled into the optical path via mirrors M6 and M7 and lenses L6 and L7, enabling visualization of the fiber facet. The reflected light from the fiber is collected by the objective and directed through beam splitter BS2 and reflected by beam splitter BS1 before being relayed by mirror M5 and lens L5 onto camera CAM1. This imaging path enables observation of the fiber facet during alignment and coupling optimization.

At the output side, the intensity is collected by a second objective (Olympus UPlanFL N

20x, NA = 0.5) and imaged onto a camera via relay optics and additional beam splitters. The setup allows recording of the transverse intensity distributions at defined distances from the hologram. Auxiliary LED illumination and dedicated imaging paths are used for alignment, inspection of the fiber facets, and verification of coupling conditions.

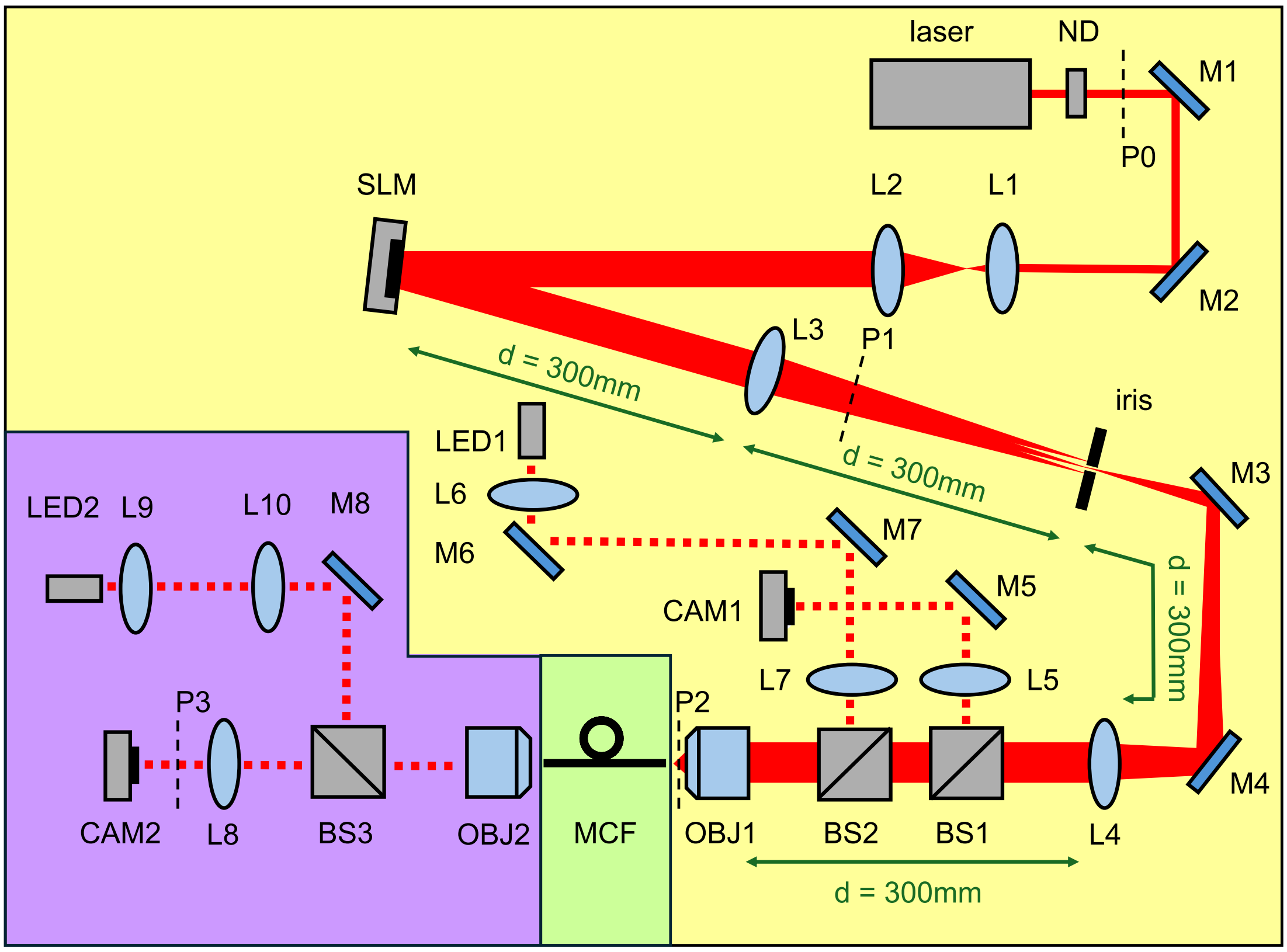

Fig. S1: Experimental setup for selective core excitation and image-plane characterization of the hologram-enhanced multicore fiber (CAM: camera, OBJ: objective, ND: neutral-density filter). The schematic highlights the three key sections of the system: the selective core excitation stage (light yellow background), the sample region containing the multicore fiber (light green background), and the intensity characterization stage (light purple background).

Sec. S 2 Coupling efficiency

To quantify the coupling efficiency and transmission characteristics, the optical power was measured at four reference positions along the optical path (black dashed lines in Fig. S1).

P0 : Optical power measured after the neutral-density filter and before the beam was directed onto the SLM, representing the input power.

P1 : Optical power measured after lens L3, corresponding to the power of the SLM-modulated beam before spatial filtering and before coupling into the fiber.

P2 : Optical power measured directly after Objective 1, representing the power incident on the fiber input facet.

P3 : Optical power emitted from the fiber output facet, determined by accounting for transmission losses from Objective 2, beam splitter 3, and lens L8.

During the coupling efficiency measurements, only a single core of the MCF was excited. To accurately assess coupling into the guided mode of a single core, the zero-order contribution was directed outside the cladding region to prevent overlap with the guided light. In addition, a partially stripped 2 m long fiber section incorporating four turns with a bending radius of approximately 6.5 cm was used. Part of this section was immersed in a higher refractive index oil to efficiently strip cladding-guided light. At the output, the emitted light was collected with Objective 2 and imaged onto the detector plane, where an aperture ensured that only light originating from the excited core reached the detector. The measured optical powers and the corresponding power ratios are summarized in Tab. S1, and the coupling efficiency into the multicore fiber was determined as η = P3/P2.

Table S1: Measured optical power levels at key positions in the experimental setup (black dashed lines in Fig. S1) and the corresponding power ratio between P3 and P2 used to determine the coupling efficiency.

P0 (μW)	P1 (μW)	P1/P0	P2 (μW)	P3 (μW)	$\eta = P_3/P_2$
112.2	66.8	0.59	10.1	4.0	0.40

Sec. S 3 Diffraction efficiency

The diffraction efficiency was determined from the recorded camera intensity distributions as follows. The image of a single generated bar (Fig. 4(a)) was converted into a grayscale intensity map and treated as a two-dimensional data array. To correct for detector background, a background level was estimated from a 200 × 200 pixel region in the top-left corner of the image, where no optical signal was present. The mean intensity of this region was subtracted from the entire image, and any resulting negative values were set to zero (Fig. S2).

The optical power in the desired diffraction order was determined by summing the pixel intensities within a rectangular region of interest (ROI) surrounding the generated bar-type intensity distribution,

$$P_{\text{beam}} = \sum_{\text{ROI}} I(x, y), \tag{1}$$

while the total optical power was obtained from the sum of all pixel intensities in the background-corrected image,

$$P_{\text{total}} = \sum_{\text{image}} I(x, y). \tag{2}$$

The diffraction efficiency was then calculated as

$$\eta = \frac{P_{\text{beam}}}{P_{\text{total}}}. \tag{3}$$

The ROI boundaries for the desired diffraction order were determined from the recorded intensity distribution after background subtraction. First, the image was smoothed with a Gaussian filter to suppress noise and small intensity fluctuations. The brightest pixel

in the smoothed image was identified and assumed to belong to the desired diffraction order. A 20 % intensity threshold was then applied to generate a binary mask of the bright diffraction features. Connected-component analysis was performed on the binary mask, and the connected region containing the brightest pixel was selected. The ROI was defined as the rectangular bounding box enclosing this region. The calculated diffraction efficiency for the selected diffraction order was approximately 18.4 %.
A similar procedure was applied to the simulated intensity distribution to estimate the theoretical diffraction efficiency. For direct comparison with the experiment, the simulation was evaluated over the same field size, and the diffraction efficiency was calculated using an equivalent region of interest enclosing the desired bar-shaped light field (Fig. S2). This analysis yields a simulated diffraction efficiency of $\approx$ 38 %, compared with the experimentally measured value of $\approx$ 18.4 %. Thus, the experimentally achieved diffraction efficiency corresponds to $\approx$ 50 % of the simulated value

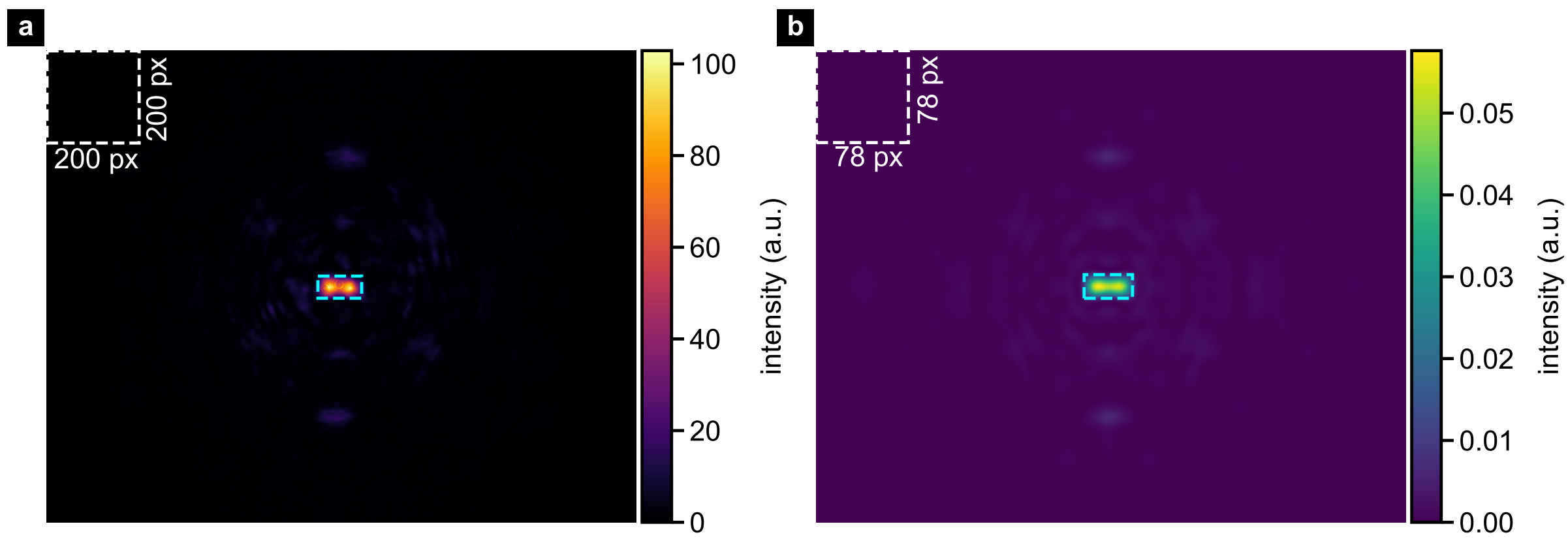

Fig. S2: Linear-scale intensity distributions used to evaluate the experimental (a) and simulated (b) diffraction efficiencies. The dashed white square indicates the background region used to estimate the average background intensity, which is subsequently subtracted from all pixel values. In the experimental image, this region comprises 200×200 pixels. Since the simulated pixel size (400 nm) is larger than the experimental pixel size (156 nm), the same physical area corresponds to 78×78 pixels in the simulation. The dashed cyan rectangle defines the region of interest corresponding to the desired diffraction order. The diffraction efficiency is calculated as the ratio of the integrated background-corrected intensity within the cyan region to the total background-corrected intensity.

Sec. S 4 Topography

The fabricated holograms were characterized by laser-scanning microscopy (LSM) to assess the fidelity of the transferred surface relief. Owing to its high lateral resolution and reliable reconstruction of steep surface gradients, LSM is well suited for characterizing the fine topographic features of the hologram. Given the designed lateral pixel size of 400 nm, the characteristic structural details are expected to be well resolved by this technique. Measurements were performed using a Zeiss LSM 800 operating at a wavelength of 405 nm and equipped with a 50x objective (NA = 0.95). The investigated structures consisted of 3D nanoprinted diffractive optical elements fabricated on fused silica substrates and coated with a 30 nm aluminum layer to ensure sufficient reflectivity for topographic imaging. The resulting measurements are summarized in Fig. S3.

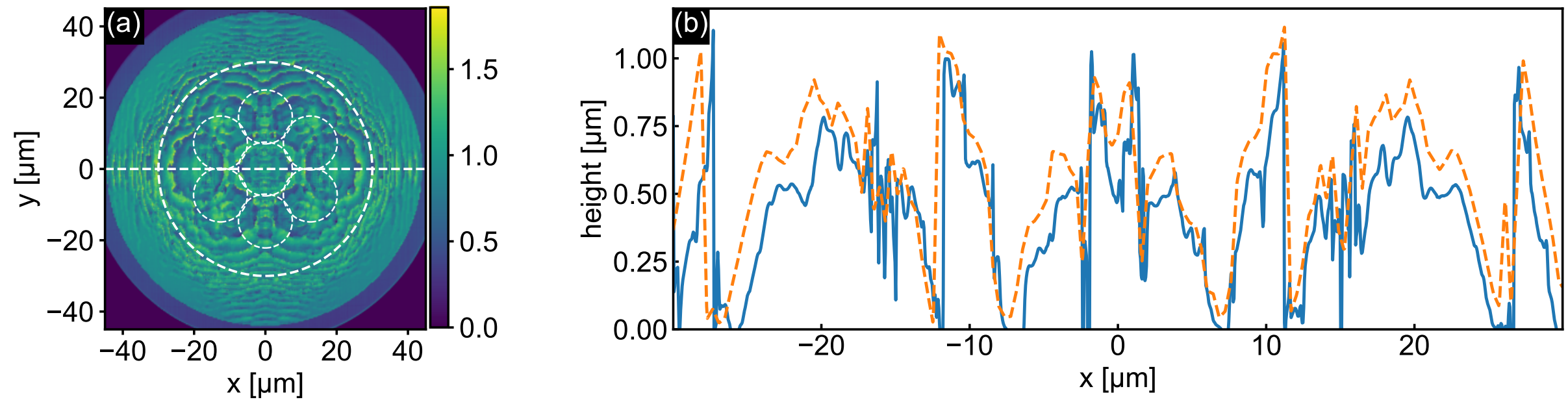

Fig. S3: Topographic characterization of the fabricated hologram and comparison with the design. (a) Laser-scanning microscopy (LSM) topography map acquired using a Zeiss LSM 800. The seven small dashed white circles indicate the projected illumination regions associated with the seven excited fiber cores, while the large dashed circle marks the optically relevant area from which the horizontal profile at y = 0 µm, shown in (b), was extracted. (b) Comparison of the measured LSM height profile (blue) with the corresponding design profile (dashed orange) along the horizontal center line of the hologram at y = 0 µm.

The LSM map (Fig. S3(a)) shows the overall geometry of the fabricated hologram, with seven small, dashed circles marking the excitation regions associated with the beams emitted by the individual fiber cores. The larger dashed circle denotes the optically relevant area from which the horizontal profile in Fig. S3(b) was extracted along the center line at y = 0 µm. This region corresponds to the primary interaction zone of the expanded beams, whereas the outer parts of the hologram contribute negligibly to the generated digit. Consequently, fabrication imperfections in peripheral areas are expected to have no significant impact on beam formation.

The extracted profile shown in Fig. S3(b) shows good agreement between the fabricated structure and the design. The positions of the main peaks and valleys closely follow the intended topography over the full 60 µm range, confirming successful implementation of the overall phase profile during fabrication. The measured profile reproduces both the low- and high-spatial-frequency features of the design, indicating that the fabrication process preserves the fine structural details required for accurate wavefront shaping. Furthermore, no systematic lateral shift, scaling error, or profile distortion is observed across the investigated region, confirming accurate transfer of the design geometry during fabrication. The most noticeable deviations are variations in the absolute height of certain features and a slight smoothing of sharp transitions in the measured profile. These effects are likely related to the fabrication process and may arise from material shrinkage during development, exposure-dose variations, finite voxel dimensions, the intrinsic low-pass filtering associated with two-photon polymerization, or the subsequent metal-coating procedure required for topographic imaging. Importantly, the relative arrangement of peaks and valleys is preserved throughout the illuminated region, indicating that the phase relationships responsible for beam shaping remain largely intact despite moderate deviations in absolute height.

Despite these deviations, the measured profiles faithfully reproduce the overall geometry, symmetry, and characteristic features of the design. The strong agreement between the measured and designed structures within the illuminated region confirms that the surface relief responsible for beam shaping was transferred with high fidelity, providing confidence that the intended optical functionality of the hologram is preserved.

Sec. S 5 z-scan of selected digit around the image plane

Supplementary Fig. S4 shows the measured transverse intensity distribution of a representative digit recorded at different propagation distances from the hologram in the vicinity of the image plane. The measurement plane was translated along the optical axis in approximately 10 µm increments, covering a range from about 140 µm to 160 µm.

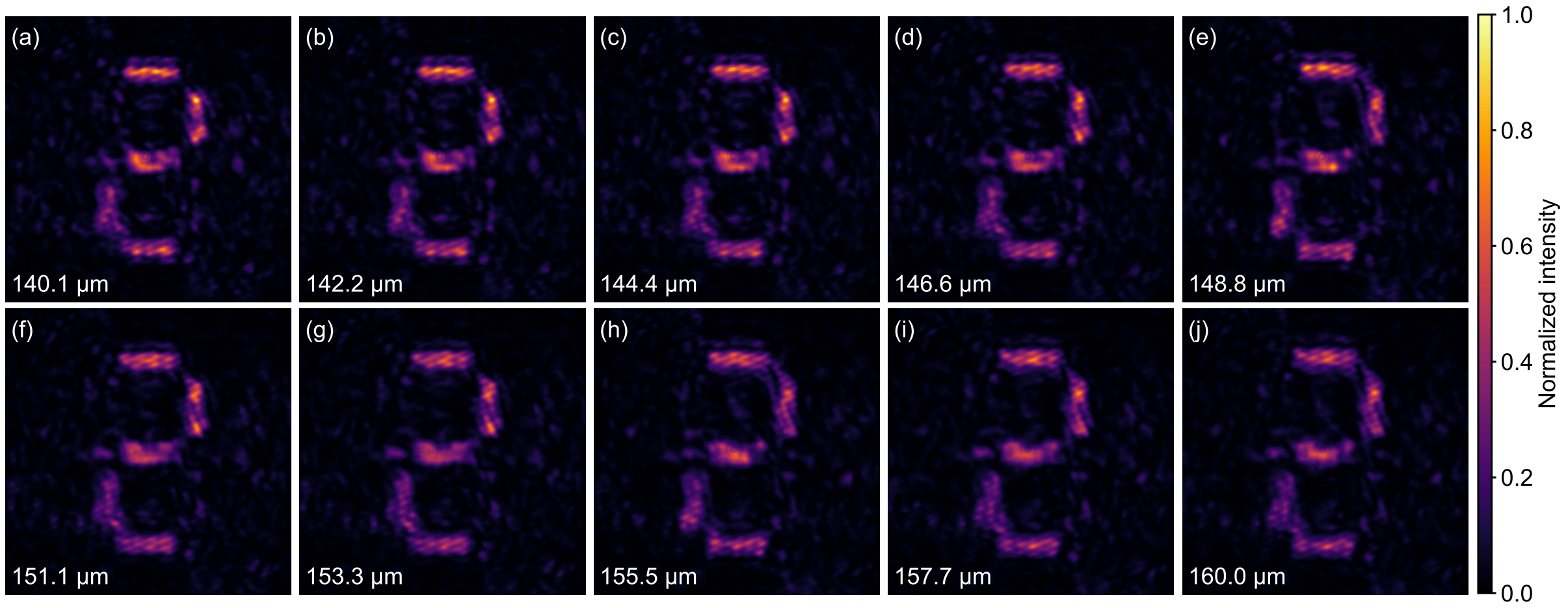

Fig. S4: Measured transverse intensity distributions of a selected digit as a function of propagation distance from the hologram. The distance values, shown in the lower-left corner of each panel, are varied in approximately 10 µm increments across the image plane. The intensity in each panel is normalized to unity.

The results reveal the clear formation of the digit within a well-defined axial region, with maximum contrast and the sharpest segment definition near the design distance of the hologram (z =150 µm). As expected, for planes located before or after the optimal image position, the digit remains recognizable but gradually blurs, with reduced contrast and increased background modulation.

Sec. S 6 Impact of Phase Variations

Statistical Analysis of the Impact of Phase Variations: To quantitatively analyze the impact of phase variations on the generated light-field patterns, we performed a correlation analysis of the holographically reconstructed intensity distributions. The purpose of this analysis is to quantify the robustness of the holographic reconstruction against random relative phase variations between the individual cores of the MCF. To remain close to the experimental situation, we analyzed the experimentally generated patterns shown in Fig. 7, each of which corresponds to a different distribution of relative phases between the excitation spots generated by the SLM-based setup, covering a phase range from 0 to 2π. We specifically employ pairwise Pearson correlation analysis, which provides a quantitative measure of the similarity between the spatial intensity distributions obtained for different random phase realizations.

We consider two reconstructed intensity images $I_i(x, y)$ and $I_j(x, y)$ obtained for two different distributions of the random inter-core phases. The Pearson correlation coefficient ρ_{ij} $(-1 \leq \rho_{ij} \leq 1)$ between the two images is defined as

$$\rho_{ij} = \frac{\sum_{x,y}\left[I_i(x,y) - \overline{I_i}\right]\left[I_j(x,y) - \overline{I_j}\right]}{\sqrt{\sum_{x,y}\left[I_i(x,y) - \overline{I_i}\right]^2}\sqrt{\sum_{x,y}\left[I_j(x,y) - \overline{I_j}\right]^2}}, \tag{4}$$

where

$$\overline{I_i} = \frac{1}{N_{\text{pix}}}\sum_{x,y} I_i(x,y) \tag{5}$$

is the mean intensity of image i, calculated over all pixels included in the analysis. Thus, the mean intensity is calculated separately for each reconstructed image and subtracted before calculating the correlation. Note that the Pearson coefficient is insensitive to an overall multiplicative scaling and an additive intensity offset. It therefore primarily quantifies the similarity of the spatial intensity distributions. For the present application, values close to unity indicate that the spatial intensity distributions obtained for two different random phase realizations are highly similar.

For N reconstructed images, the Pearson coefficients are calculated for every pair of images, leading to the correlation matrix

$$\mathbf{C} = \begin{pmatrix} 1 & \rho_{12} & \rho_{13} & \cdots & \rho_{1N} \\ \rho_{21} & 1 & \rho_{23} & \cdots & \rho_{2N} \\ \rho_{31} & \rho_{32} & 1 & \cdots & \rho_{3N} \\ \vdots & \vdots & \vdots & \ddots & \vdots \\ \rho_{N1} & \rho_{N2} & \rho_{N3} & \cdots & 1 \end{pmatrix}. \tag{6}$$

Since $C_{ii} = 1$ and $C_{ij} = C_{ji}$, only one triangular part of the matrix contains the independent pairwise comparisons.

Applying this analysis to seven representative experimental realizations shown in Fig. 7 yields the correlation matrix presented in Fig.8(a). High pairwise Pearson correlation coefficients are obtained throughout the matrix, with ρ_{ij} ranging from approximately 0.77 to 0.92 for the displayed realizations.

Furthermore, to obtain a more comprehensive statistical assessment, all 15 experimentally recorded realizations shown in Fig. 7 were considered, corresponding to 105 independent pairwise comparisons. The resulting distribution of Pearson correlation coefficients is shown in Fig. 8(b).

Impact of Fiber Bending on Intermodal Phase: To quantitatively assess the impact of fiber bending on the relative phase between the beams interfering in the hologram plane, additional fiber-bending experiments were performed using the procedure outlined in Ref. [1]. In these experiments, a specific pair of MCF cores was simultaneously excited with an initial relative phase difference of zero, and the resulting interference pattern was recorded at a defined distance from the fiber end face while the fiber was bent. For this purpose, the measurement and data-processing procedure described in Ref. [1] was implemented by mounting part of the imaging setup on a vertical translation stage while keeping the front section fixed (Fig. S5(a)). By vertically displacing the stage, a controlled tilt of the MCF, characterized by the angle θ, was introduced. As a representative example, the core pair highlighted in Fig. S5(b) was addressed. This configuration was selected because a pronounced bending-induced phase difference is expected for bending in the yz-plane.

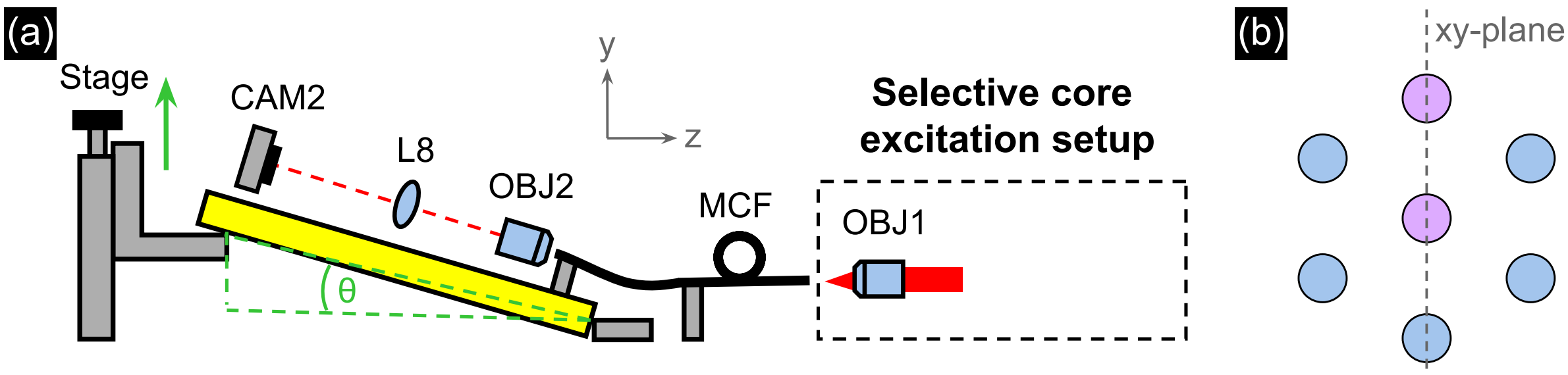

Fig. S5: Experimental setup for investigating bending-induced inter-core phase variations in the MCF. (a) Schematic of the bending setup. One side of the imaging system is mounted on a vertical translation stage, allowing a controlled MCF tilt by the angle theta while the interference pattern of two selected cores is recorded using CAM2. (b) Cross-sectional arrangement of the MCF cores, with the two simultaneously excited cores highlighted in purple. The selected core pair is aligned along the yz-plane, corresponding to the plane in which fiber bending is introduced.

The experimentally recorded interference patterns for increasing fiber-bending angles are shown in Fig. S6. A clear displacement of the interference fringes relative to the fixed horizontal reference line (yellow dashed line) is observed with increasing bending angle. This systematic displacement directly indicates a change in the relative phase between the modes at the MCF output experimentally confirming that bending of the MCF induces a relative phase shift between the individual core.

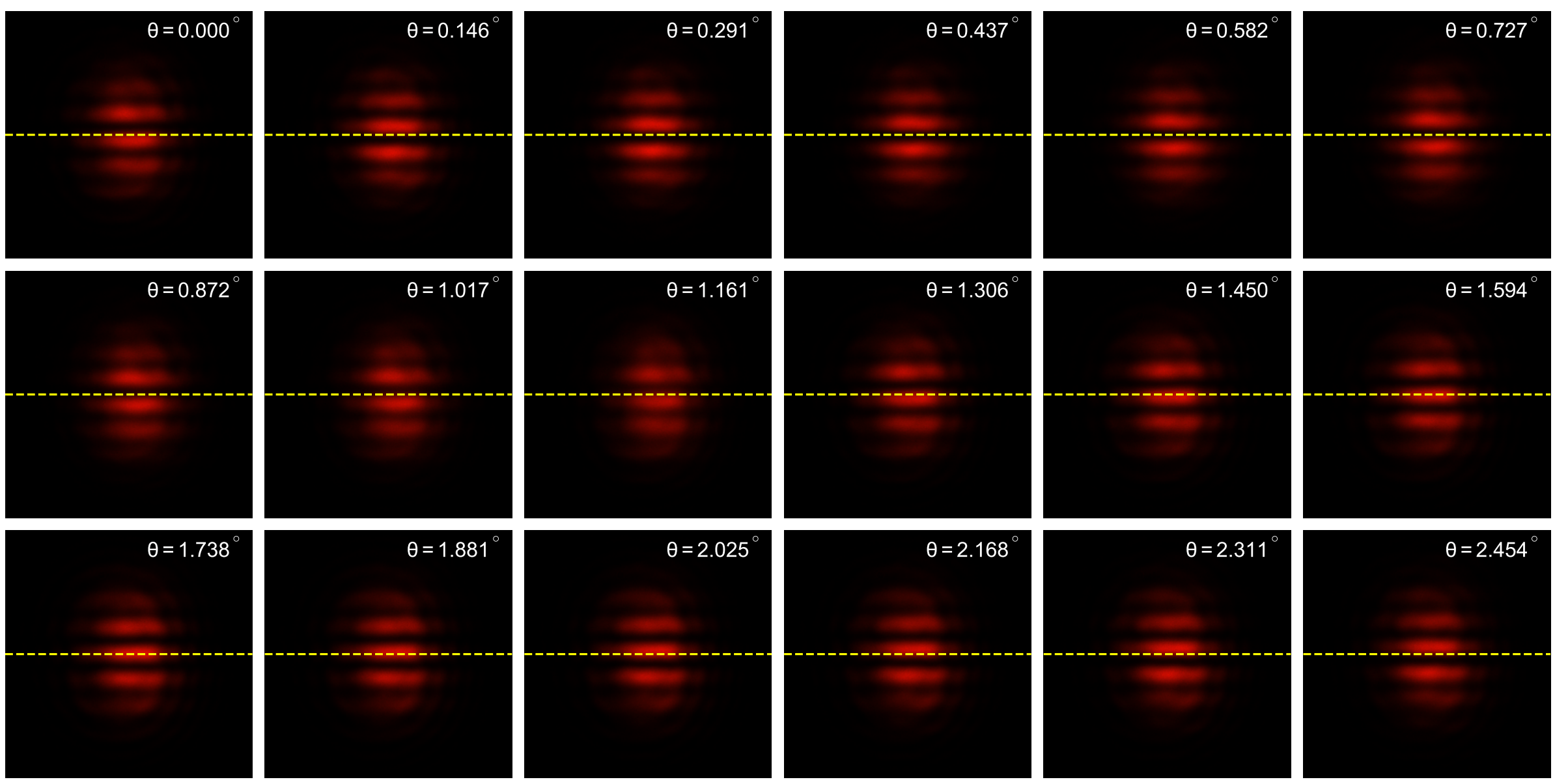

Fig. S6: Experimentally recorded interference patterns for increasing fiber-bending angles. The systematic displacement of the interference fringes with increasing bending angle indicates a bending-induced change in the relative phase between the two selected MCF cores. The yellow dashed line serves as a guide to the eye for visualizing the fringe displacement.

To retrieve the relative phase $\Delta\phi$ between the two beams, each recorded interferogram was analyzed using a two-dimensional fast Fourier transform following the procedure described by McBride et al. [1]. The interference fringes produce a pair of conjugate sidebands in the spatial-frequency domain, whereas the slowly varying intensity envelope contributes primarily to the central DC component. One sideband was consistently selected for the

entire image series, and its complex Fourier amplitude was evaluated within a fixed region around the corresponding peak. The phase of this complex amplitude provides the relative optical phase between the two interfering core beams, which was subsequently unwrapped to clearly trace the phase evolution. The resulting phase difference exhibits an approximately linear increase with increasing bending angle, as shown by the experimental data in Fig. RL5 (green dots). This behavior is consistent with the analytical relation reported by McBride et al. [1], according to which the bending-induced phase difference between two cores is given by

$$\Delta\phi = 1.16\left(\frac{2\pi d}{\lambda_0}\right)\theta + \phi_0 \tag{4}$$

where d is the separation between the two excited cores, λ_0 is the wavelength, θ is the bending angle, and ϕ_0 is the initial relative phase offset. For the parameters of the present experiment ($d = 14.6\,\mu\mathrm{m}$, $\lambda_0 = 637\,\mathrm{nm}$), this relation predicts a phase sensitivity of approximately $2.9\,\mathrm{rad}/^\circ$ (dashed green line in Fig. S7). The measured phase evolution follows this theoretical prediction closely over the investigated angular range, confirming that even relatively small bending angles induce significant changes in the relative phase between two MCF modes.

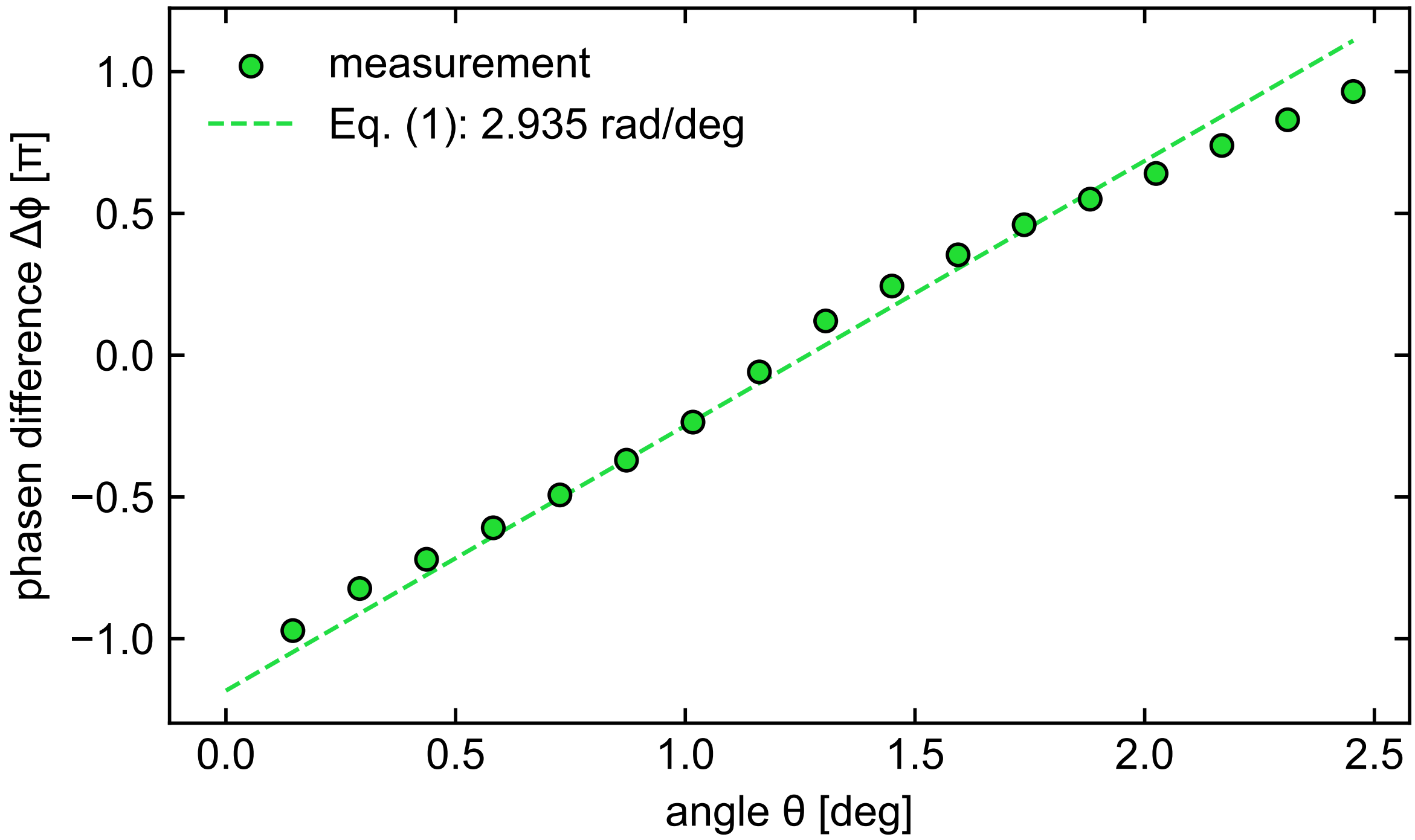

Fig. S7: Bending-induced relative phase difference between the two selected MCF cores as a function of the fiber-bending angle. The green dots show the experimentally retrieved phase differences, while the dashed green line represents the analytical prediction according to McBride et al. [1], yielding a phase sensitivity of $2.9\,\mathrm{rad}/^\circ$ for the parameters of the present experiment.

Sec. S 7 Two cores interference

The accuracy of the applied phase control was verified by checking whether a defined phase shift imposed at the input of a selected core via computer control is preserved

at the fiber output. Fig. S8 shows the interference patterns of two selectively excited cores recorded at different propagation distances from the fiber facet, revealing a clear two-beam interference pattern with well-defined fringes.

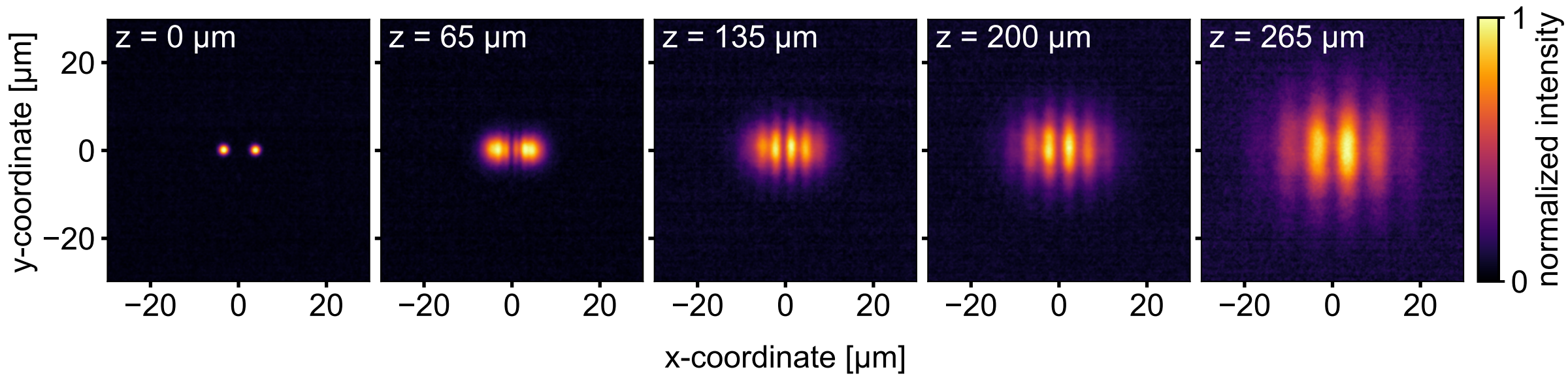

Fig. S8: Interference patterns from two selectively excited fiber cores (relative phase $\Delta\Phi = 0$) recorded at different propagation distances from the fiber facet (indicated by the top-left numbers). Each image has been normalized to its maximum value.

In addition, the interference pattern at a distance of 265 µm from the facet is shown in Fig. S9. It is observed that introducing a relative phase shift of $\Delta\Phi = \pi$ between the modes in the two cores results in a clear shift of the interference pattern, with a previously bright fringe replaced by a dark fringe. These results confirm coherent excitation and reliable phase transfer through the multicore fiber.

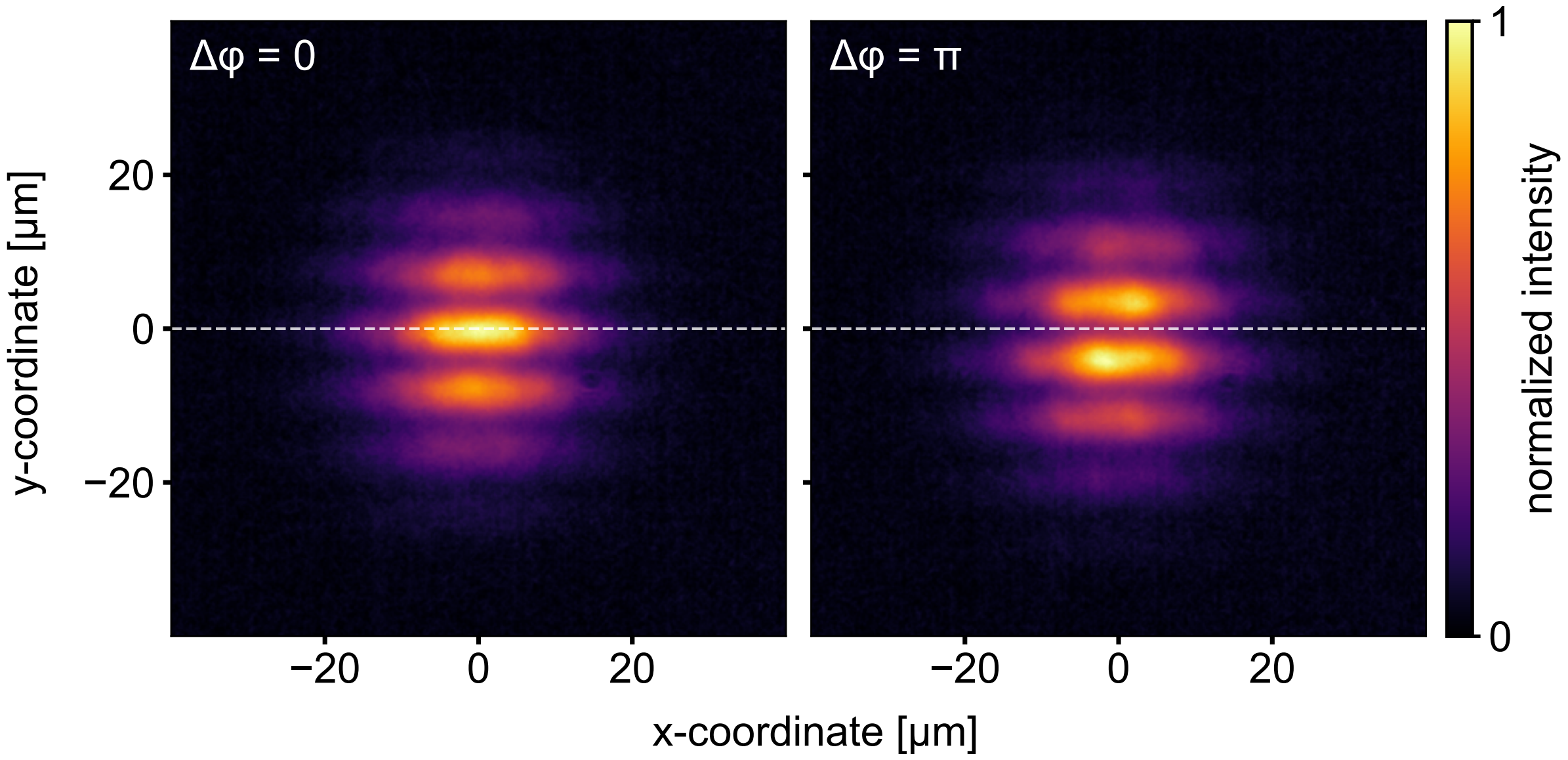

Fig. S9: Interference pattern of two selectively excited cores recorded at a propagation distance of 265 µm from the fiber facet at different relative phases ((a) $\Delta\Phi = 0$ and (b) $\Delta\Phi = \pi$). Each image has been normalized to its respective maximum. The horizontal dashed white line indicates the maximum intensity for $\Delta\Phi$=0.

Sec. S 8 Phase-amplitude modulation

To verify that the phase modulation applied by the SLM does not affect the amplitude of the excited mode, the output of a single excited core was recorded at a distance of 100 µm from the hologram while systematically varying the imposed input phase at a constant amplitude. The corresponding intensity distributions are shown in Fig. S10, and the extracted average normalized intensity is presented in Fig. S11.
It is observed that the intensity remains essentially constant across the full phase range (average normalized intensity 18.5 %, relative standard deviation 3 %), with small residual fluctuations attributed primarily to air turbulence and minor mechanical drift in the laboratory environment. These results confirm that the applied phase shift does not affect the amplitude, demonstrating the absence of phase-amplitude coupling and invariance under global phase variation.

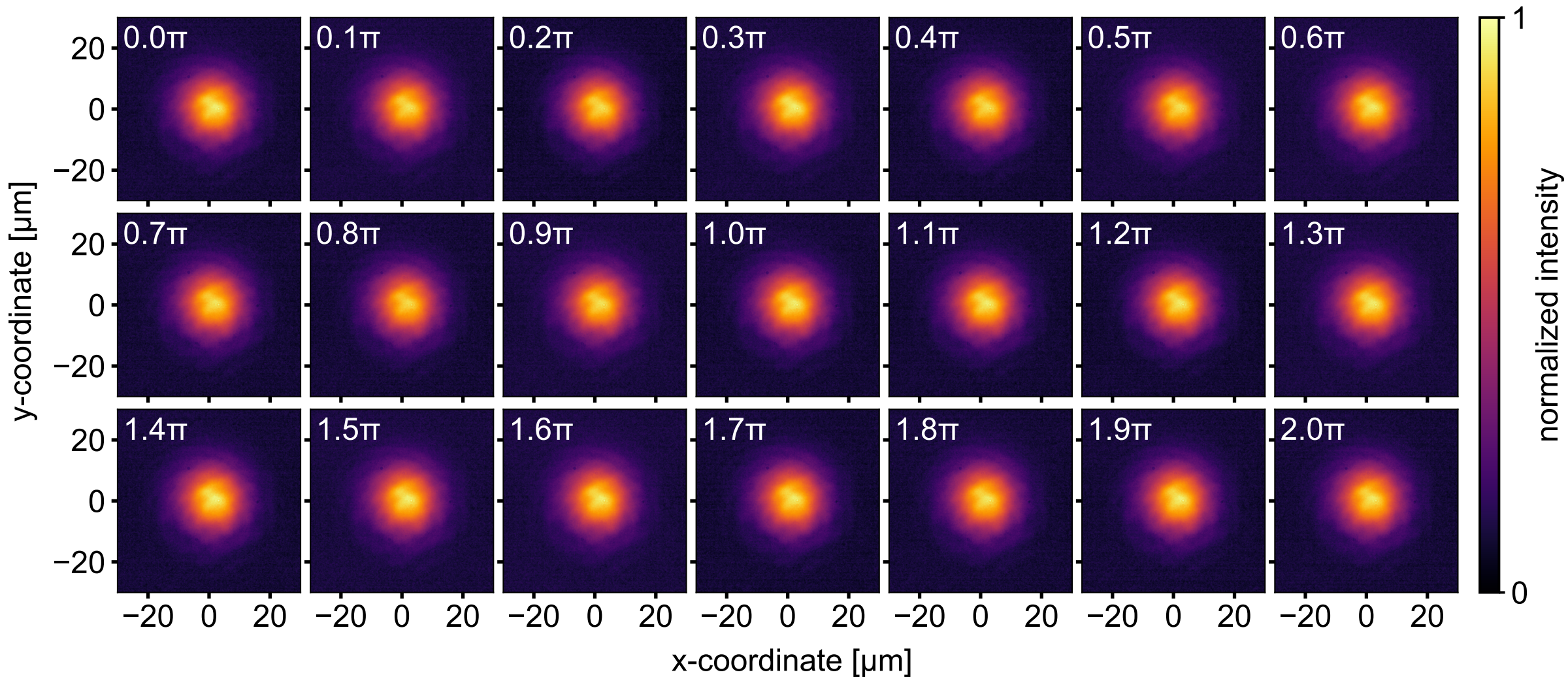

Fig. S10: Output intensity distributions of a single excited core recorded at a propagation distance of 100 µm from the hologram for different input phase values applied via the SLM, with all other parameters held constant. The results confirm that the intensity profile is invariant under global phase variation. All images are normalized to the global maximum intensity across the entire dataset, so that each pixel value is divided by the highest intensity observed in any recorded image. Consequently, the brightest pixel across the full series is set to 1 while preserving the relative intensity differences between phase settings.

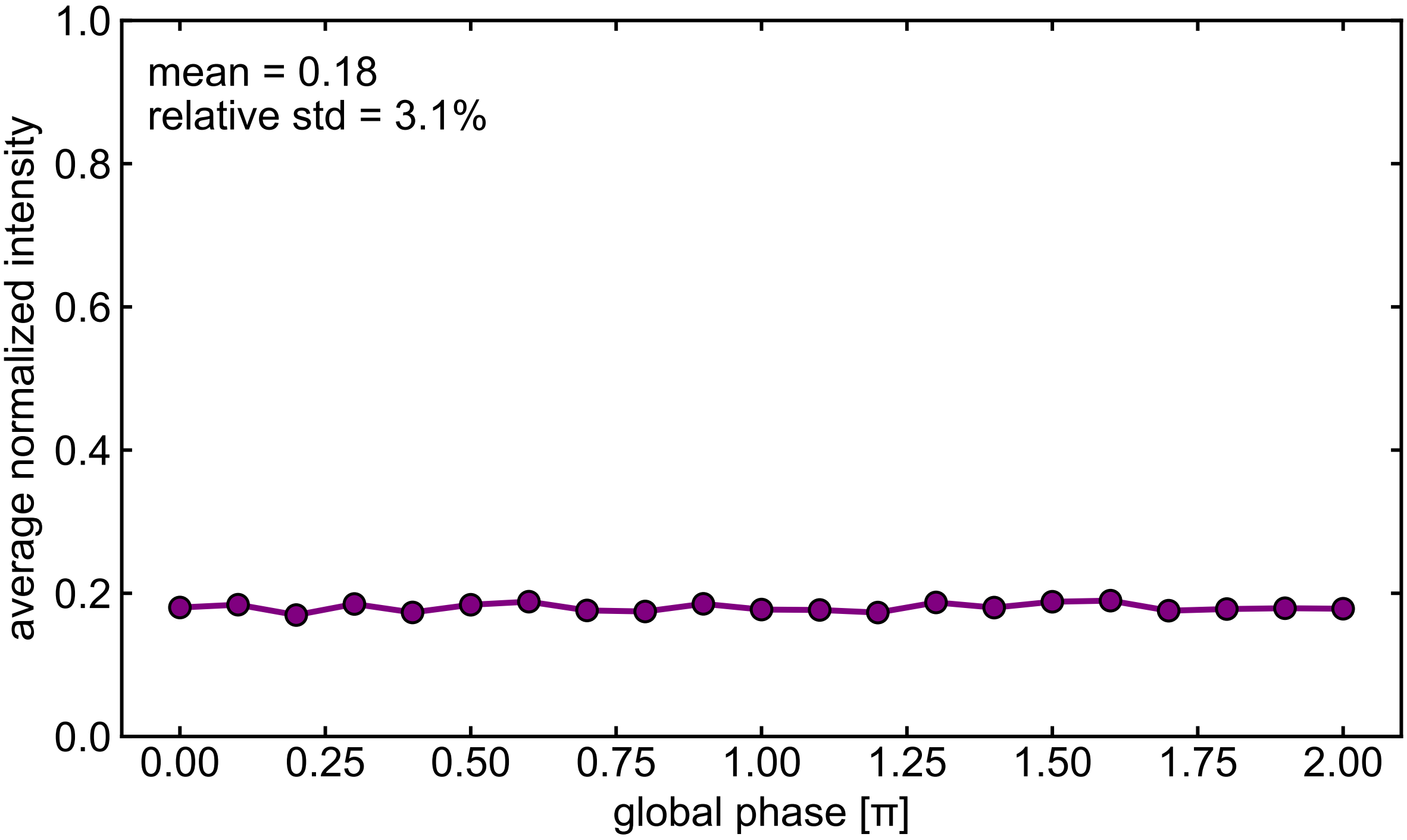

Fig. S11: Average normalized intensity within each image shown in Fig. S10 as a function of the applied input phase, demonstrating a constant amplitude and the absence of phase-amplitude coupling.

References

[1] MJ Gander, EAC Galliot, R McBride, JDC Jones, JG Burnett, and AH Greenaway. Bend measurement using multicore optical fiber. In *Optical Fiber Sensors*, page OWC6. Optica Publishing Group, 1997.
